# O_2_ Reduction Stimulates Adatom Generation on Cu(111) Catalyzing Hydrogen Evolution

**DOI:** 10.1021/jacs.5c20244

**Published:** 2026-02-11

**Authors:** David Raciti, Zisheng Zhang, Ally Guo, Thomas P. Moffat

**Affiliations:** † Materials Science and Engineering Division, 10833 National Institute of Standards and Technology, Gaithersburg, Maryland 20899, United States; ‡ SUNCAT Center for Interface Science and Catalysis, SLAC National Accelerator Laboratory, Menlo Park, California 94025, United States; § Department of Chemical Engineering, 6429 Stanford University, Stanford, California 94305, United States

## Abstract

Electrochemical mass spectrometry (EC-MS) was used to investigate the coupled dynamics of surface hydride formation, the oxygen reduction reaction (ORR), and the hydrogen evolution reaction (HER) on Cu(111) in perchloric acid. Starting with an Ar-saturated electrolyte, hydride formation proceeds via two overlapping cathodic waves that evolve with cycling due to the restructuring of the electrode surface, associated with the removal of residual oxide species. Grand canonical free-energy calculations indicate that the surface hydride stabilizes pristine terraces against roughening and helps to anneal vacancy-adatom defects introduced during specimen preparation. Introducing controlled amounts of O_2_ markedly perturbs this behavior, shifting hydride formation to more negative potentials and accelerating HER kinetics, as revealed by EC-MS. Density functional theory and molecular dynamics simulations show that coadsorption of H with ORR intermediates (OH*/OOH*) promotes Cu(111) restructuring through adatom–vacancy formation and subsurface O incorporation. The resulting fluxional adatom sites enhance the HER activity and modulate the ORR kinetics under mixed control. Extended O_2_ exposure irreversibly restructures the surface and reshapes the hydride formation waves resulting in a lasting imprint on surface reactivity that remains even after returning to nominally O_2_-free conditions. These findings demonstrate that coupled adsorbates restructure Cu(111) under an electrochemical bias, generating new active sites with direct implications for the performance and stability of Cu electrocatalysts.

## Graphical abstract

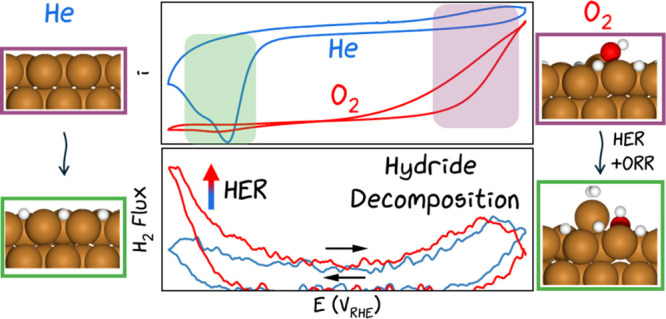

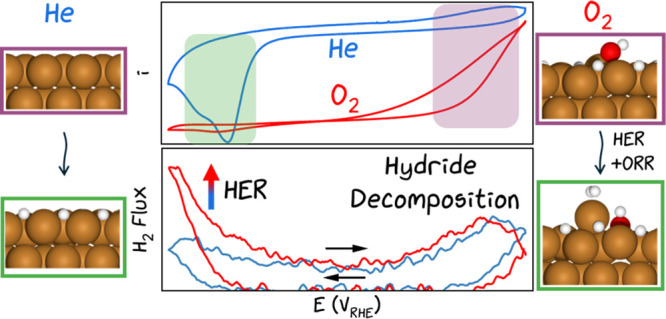

## Introduction

Reactions between Cu and O_2_ are ubiquitous in many industrially important applications, ranging from corrosion and chemical mechanical planarization to electrocatalysis. The first case is a consequence of undesired exposure to the ambient, while for the others introduction of O_2_ during processing or catalysis is central to the desired outcome. Among the different possible configurations, oxide-derived copper (OD-Cu) has emerged as a leading electrocatalyst for the conversion of CO_2_ to value-added products. The elevated catalytic performance being linked to reduction of parent copper oxides, which creates defect-rich, undercoordinated Cu surface sites with enhanced CO adsorption properties.^1–4^ The role of residual surface or subsurface oxygen in such reactions is widely debated and several studies indicate that trace O_2_ can promote CO_2_ reduction selectivity.^5–9^ Comparatively, little is known about H adsorption on such defect-laden surfaces, particularly under reaction conditions. Accordingly, investigating competitive and collaborative interactions between H, C, and O containing-adsorbates as well as their reaction intermediates, under applied potential and varying defect structures will be central to understanding the catalytic behavior of Cu catalysts toward hydrocarbon production.^1,10^

Copper–hydrogen interactions on Cu surfaces have been examined in both vacuum and electrochemical environments, revealing two-dimensional hydride superlattices and competitive adsorption with CO.^11–22^ Electrochemical scanning tunneling microscopy has shown that, in sulfuric acid, a series of (4 × 4) hydride overlayers form on Cu(111), with hydrogen (H_ads_) coverage increasing with overpotential.^11^ Voltammetric asymmetry in both potential and charge is linked to slow hydride formation by proton reduction, while desorption proceeds primarily through H_ads_ recombination, with both processes influenced by anion adsorption.^11–14^ In the absence of strongly adsorbing anions, hydride formation yields a surface coverage of ≈0.8 ML hydride in 0.1 mol L^–1^ HClO_4_, which decomposes via two pathways, recombination to H_2_ or oxidation back to H^+^—the latter only proceeding competitively above the reversible hydrogen equilibrium potential (RHE).^23^ Interestingly, at potentials sufficiently negative to produce H_ads_ > 0.75 ML, the Cu surface undergoes restructuring that generates ordered adatom sites, which are catalytically active for the hydrogen evolution reaction (HER).^11^

To explore the role of oxygen species in hydrogenation reactions, the interactions between the hydride surface and the oxygen reduction reaction (ORR) merit closer examination. Previous ORR studies on Cu in sulfuric acid revealed that sulfate adsorption acts as a site-blocking agent that inhibits the onset of the ORR.^24^ Selective two-electron reduction of O_2_ to H_2_O_2_ occurs on Cu(111), while four electron reduction to water dominates on Cu(100).^24^ Interestingly, the potential-dependent selectivity toward peroxide production on Cu(111) overlaps with that of hydride formation, suggesting a possible mechanistic connection between these two processes—an observation reminiscent of the transition to peroxide generation on H_upd_ covered Pt(111) surfaces.^25^ In contrast, the relationship between hydride formation and ORR product selectivity on Cu in perchloric acid electrolyte is less straightforward. In the absence of strong anion adsorption, oxygen and its reduction intermediates interact with Cu(111) at more positive potentials. Comparing a prior rotating-ring disc electrode study of ORR on Cu(111) with other literature findings on the corrosion of Cu single crystal surfaces suggests there is a significant overlap between H_2_O_2_ production and the equilibrium Cu^+^ concentration otherwise seen in the absence of O_2_.^24,26,27^ This is congruent with a scavenging homogeneous cross-reaction between O_2_ and Cu^+^ to produce peroxide, although connection to the rotating ring disk electrode (RRDE) results has not been explicitly made.^24^ Accordingly, a closer examination of the interactions between Cu/Cu^+^/Cu^2+^, anion adsorption, ORR, surface hydride formation, and their collective impact on hydrogenation reactions is warranted.

In this study, the competitive and cooperative surface interactions between hydride formation and decomposition, hydrogen evolution and oxidation, and the ORR on Cu(111) in 0.1 mol L^–1^ HClO_4_, are examined by using electrochemical mass spectrometry (EC-MS). Exposure to controlled amounts of O_2_ led to an unanticipated increase in the hydrogen evolution reaction rate. Following ORR exposure, the two voltammetric peaks associated with hydride formation, in deaerated 0.1 mol L^–1^ HClO_4_, exhibit altered peak currents while exposure to controlled amounts of O_2_ leads to a monotonic increase in the HER rate. Grand canonical (GC) methods, density functional theory (DFT) calculations, and molecular dynamics (MD) simulations indicate that the increased rate is due to fluxional interactions between ORR reaction intermediates and the hydride surface, leading to thermodynamically and kinetically favorable self-roughening of the surface via the reversible and irreversible formation of adatom moieties. The resulting undercoordinated adatoms contribute to the enhanced HER observed by EC-MS in the presence of O_2_. Furthermore, the formation and decomposition of the hydride surface after sustained O_2_ exposure are measurably distinct from those in the nominally O_2_-free case.

## Methods

### Preparation of Cu(111) or Pt Polycrystalline Electrode

The preparation of the metal crystals have been previously discussed.^14^ In brief, the electrodes were mechanically polished down to a 0.05 μm alumina finish followed by several cycles of rinsing, with 18.2 MΩ·cm H_2_O, and sonication to remove residual alumina particles. For calibration measurements, a Pt disk electrode (5 mm diameter) was flame annealed using a H_2_ torch for ≈1 min, cooled to room temperature, and then loaded into the EC-MS headpiece for potential pulse experiments. Prior to surface hydride studies the Cu(111) disk (5 mm diameter) encapsulated in a Pine Instruments* Teflon u-cup was electrochemically polished at 1.6 V vs a Pt counter electrode using concentrated phosphoric acid for 5 min. The Cu(111) surface was polished with the exposed surface facing upward in the electropolishing cell with the larger area Pt gauze counter electrode located ≈6 cm away. The Cu(111) disk electrode was then thoroughly rinsed, protected with H_2_O, transferred to the EC-MS headpiece, finally dried with Ar, and immediately mounted to a mass spectrometer.

### Electrochemical Mass Spectrometry Measurements

EC-MS was performed using a thin-layer cell configuration, as developed by Spectroinlets.^28,29^ A freshly prepared Ar purged 0.1 mol L^–1^ HClO_4_ electrolyte (70% HClO_4_, 99.999% trace metals basis from Sigma-Aldrich) was used in all measurements. The two glass side arms on either side of the working electrode contained an Ir wire counter electrode and a trapped H_2_ bubble reversible hydrogen reference electrode, respectively. The latter was configured as a Pt wire immersed in a H_2_ saturated electrolyte enclosed in a glass tube with a fine capillary, providing continuity to the electrolyte in the main cell. The Ir wire counter electrode was positioned over 3 cm away from the working electrode compartment to minimize any effects of the counter electrode reactions. To prevent the oxidation and dissolution of the working electrode, 0 V_RHE_ (assume RHE throughout) was applied immediately after the three electrodes made contact with the electrolyte. During idle periods between EC-MS measurements, the working electrode was maintained at 0.175 V. To investigate the ORR reaction, O_2_ was added to the He carrier gas, the latter serving to pressurize the MS sampling chamber, whereupon the gases dissolve across the membrane pores into the electrolyte in the working electrode compartment in accord with Henry’s law.

Mass spectrometry was performed by using a Pfeiffer PrismaPro QMG-250 quadrupole mass spectrometer with a Faraday cup detector. Electron multiplier operation was disabled during these measurements. Single ions were monitored based on the experiment; typically 2, 4, and 32 amu were acquired with a dwell time of 64 ms per mass unit. The ionizer energy was set to approximately 70 eV.

The steady-state O_2_ concentration in the sampling chamber was confirmed by monitoring 4 and 32 amu, occurring within ≈10 min of starting the O_2_ gas flow. A Biologic SP-200 was used for all electrochemical measurements. EC-MS and electrochemistry data were collected on the same computer enabling accurate time synchronization between the two measurements. For cyclic voltammetry measurements the mass spectrometry baseline signal was subtracted using a linear profile, with the slope determined by averaging the spectrometry signal over 2 s before and after completing the voltammetric scan.

For potential step measurements, the baseline was zeroed via averaging the 2 amu signal for ≈2 s before initiating each cathodic potential step (*E*_Pulse_). Detailed calibration procedures for determining the H_2_ flux from the 2 amu peak, especially in the presence of O_2_, is provided in the supplementary section “Calibration of the EC-MS instrument for H_2_ quantification in varying carrier gas mixtures.” (Figures S1–S3). The electrolyte thickness in the working electrode compartment was determined, at the conclusion of the experiment, using an impulse procedure discussed previously and was found to be ≈120 μm (Figure S4).^14,30^

### Computational Details

The copper surface is modeled by a 4-layer 4 × 4 supercell with respect to Cu(111) 1 × 1 lattice termination, with a surface area of 90.532 Å^2^. The bottom 2 atomic layers of the slab are constrained as the bulk region, and all others are allowed to relax as the interface region. A vacuum slab of 15 Å thickness is added in the surface normal direction to avoid spurious interactions between periodic images. The DFT calculations are performed with the Revised Perdew–Burke–Ernzerhof (RPBE) functional and Perdew–Burke–Ernzerhof projector augmented wave (PBE_PAW) pseudopotentials using the Vienna Ab initio Simulation Package (VASP) program (version 5.4.4).^31–35^ The choice of pure RPBE without dispersion correction has been demonstrated by our previous computational benchmarking and joint experimental studies to be the overall optimal setting in balancing computational cost, adsorption geometry, and energetics for Cu surfaces under electroreduction conditions.^1,36–39^ The convergence criteria for electronic and force minimization are 10^–5^ eV and 2.0 × 10^–2^ eV/Å, respectively. The electronic convergence criterion is tightened to 10^–6^ eV for evaluating the final energies. The Brillouin zone is sampled by a single Γ point due to the very large number of configurations in the sampling process. The kinetic energy cutoff for the plane waves is 400 eV during minima searches and molecular dynamics simulations, and 500 eV in the final energy evaluation. The testing of the number of atomic layers, kinetic energy cutoffs, *k*-point(s), and convergence criteria is provided in Figures S27–29.

The global optimization minima searches are performed using our open-source Python package, grand canonical global optimizer for clusters, interfaces, and adsorbates (GOCIA).^40^ The surface phase of the pristine Cu(111) under varying H coverage is sampled using the grand canonical genetic algorithm (GCGA) on the basis of the samples in Cheng et al.^11^ For the roughened case, we employ the constrained GCGA technique, using a roughness threshold to bias the search toward the metastable roughened surface phases.^41^ The most stable adsorption configurations of OH and OOH on the hydride surfaces are searched using the box-sampling method implemented in GOCIA. Note that the model herein represents an ordered crystalline surface sample stabilized under hydride-forming, acidic reducing conditions (vide supra), where hydride dominates the surface chemistry, and oxygen-containing species are expected to be transient and minimal.^13,14,23^ This differs from oxide-derived Cu systems, which involve less crystalline structures with persistent oxygen remnants.^42^ Our computational study therefore focuses on a distinct hydride-centric crystalline Cu regime relevant to our experiments.

To better account for the realistic factors at the aqueous electrochemical interface, we model the polarizable electrolyte using the linearized Poisson–Boltzmann model as implemented in the implicit solvation code VASPsol.^43^ The potential-dependent electronic free energies Ω are calculated using the GC-DFT scheme^44^ on symmetrized slabs by the surface charging method^45^ implemented in GOCIA, assuming a constant interfacial capacitance.

The ab initio molecular dynamics (AIMD) simulations are performed using the same DFT methods (vide supra) at 300 K within the *NVT* ensemble with the Nose–Hoover thermostat and a time step of 1 fs.^46,47^ To obtain the free energy profile of the pristine-to-roughened transition of the surface phase, we constrain the height of the adatom Cu as the roughening coordinate and sample along it using the slow-growth technique.^48–50^ To be specific, the roughening coordinate is varied by 0.00025 Å per fs during the simulation so that all other degrees of freedom are sufficiently equilibrated. The final free energy surface is obtained by thermodynamic integration of the constraint force along the roughening coordinate.

## Results and Discussion

### Voltammetric Measurements

Hydride formation involves two potential-dependent processes, as evidenced by the two cathodic peaks observed in perchloric acid (Figure 1). An analysis of the charge and 2 amu EC-MS signals (i.e., H_2_ flux) indicates that the transition between the two waves corresponds to an H_ads_ coverage of ≈0.65 to 0.8 ML. This coverage aligns closely with the threshold identified by recent computational surface phase diagrams, beyond which significant electrode restructuring and the onset of substantial HER activity occur.^11,23^

**Figure 1:**
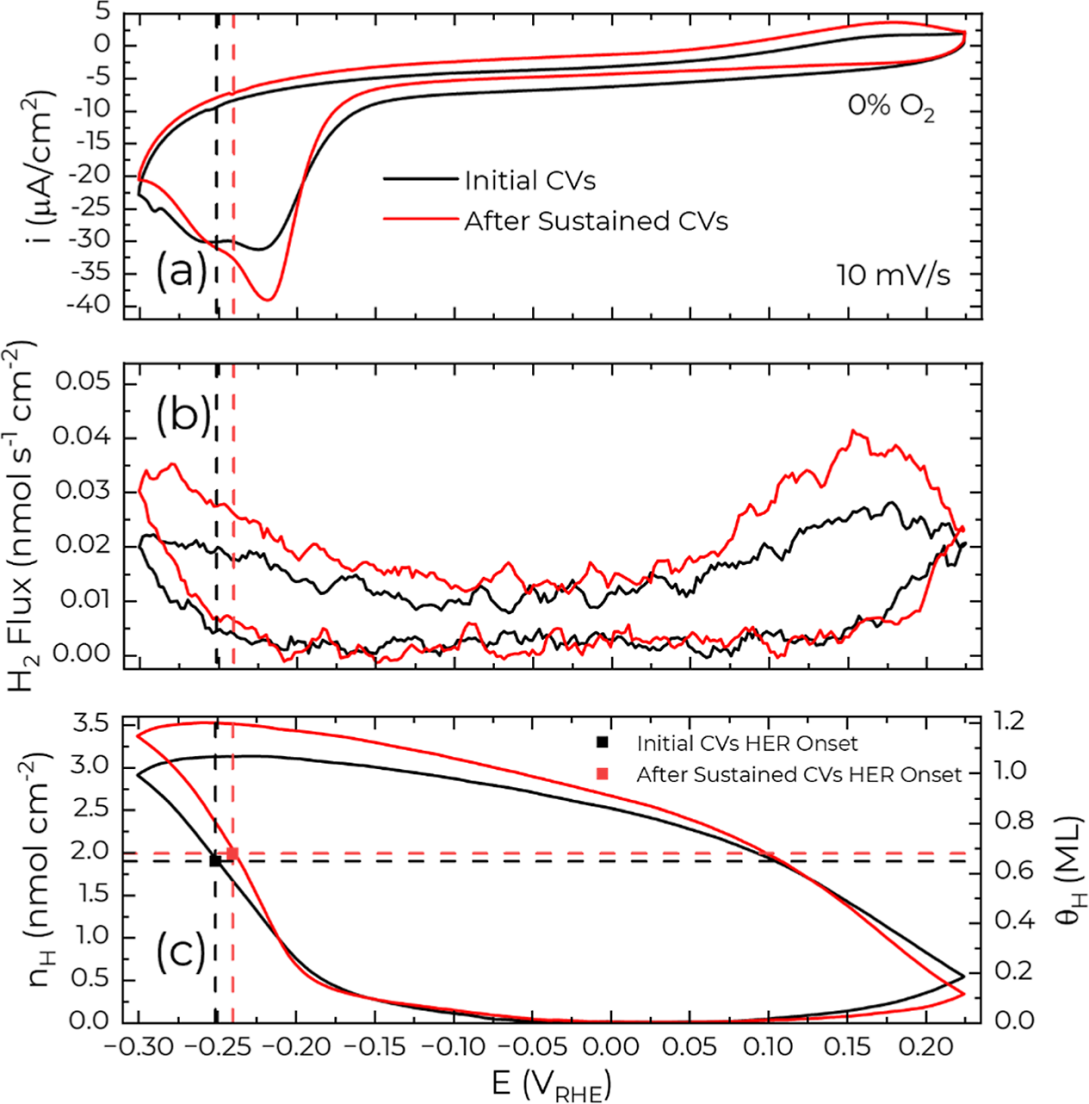
Tracking the change in (a) voltammetry and (b) 2 amu mass spectrometry (i.e., H_2_ flux) features associated with hydride formation on Cu(111) in He saturated 0.1 mol L^–1^ HClO_4_. The “initial CVs” voltammetric cycle was collected in the first batch of cycles after starting the EC-MS experiment and is labeled scan 5 in Figure S5. The voltammetric cycle denoted “after sustained CVs” was obtained following potential pulse measurements in the He saturated electrolyte, to be discussed below, and is denoted scan 14 in Figure S5. The H_2_ flux in (b) was smoothed using 5-point adjacent averaging. (c) Estimated H_ads_ on the Cu(111) surface by subtracting the integrated H_2_ flux from the charge under the assumption that all charge is related to H reduction or oxidation. Figure S6 provides more detail on how (c) was obtained.

The relative magnitudes of these peaks evolve during EC-MS measurements in He saturated 0.1 mol L^–1^ of HClO_4_ (Figure 1a), reflecting path-dependent electrode restructuring. Initial voltammetry, conducted immediately following electropolishing the Cu(111) surface and assembling the EC-MS cell, reveals nearly equivalent peak currents. Subsequent cycling progressively enhances the first peak relative to the second (Figure S5). Similarly, voltammetry performed after a series of cathodic potential pulses in the He saturated electrolyte (Figure 1a “after sustained CVs”) demonstrates an increase in the first peak relative to the second, which again speaks to ongoing surface reconstruction with cycling.

Additionally, prolonged cycling leads to a reduction in the voltammetric background current, likely attributable to the gradual removal of residual dissolved oxygen introduced during cell assembly and electrolyte preparation. For instance, at 0 V, the current density decreases from −6.3 μA cm^–2^ during initial cycling (“initial CVs,” Figure 1a) to −4.3 μA cm^–2^ after sustained cycling. This reduction suggests residual O_2_ levels, which decreases slowly over time, influence both hydride formation and Cu surface restructuring.

Corroborating these observations, EC-MS measurements demonstrate enhanced HER kinetics with sustained cycling (Figure 1b). Evaluating the maximum achievable H_ads_ coverage (see Figure S6 for details) reveals that HER onset consistently follows the establishment of fractional hydrogen coverage between ≈0.67 and 0.8 ML as indicated by Figure 1c, with the exact value being scan rate dependent as is evident in Figures 1 and 2 in ref 23. As some portion of the integrated charge goes to the capacitive charging, the so-determined coverage values represent an upper bound for H_ads_ coverage. Of further interest, the continuous evolution of the electrode structure with cycling leads to a +10 mV shift in the HER onset relative to initial scans.

**Figure 2:**
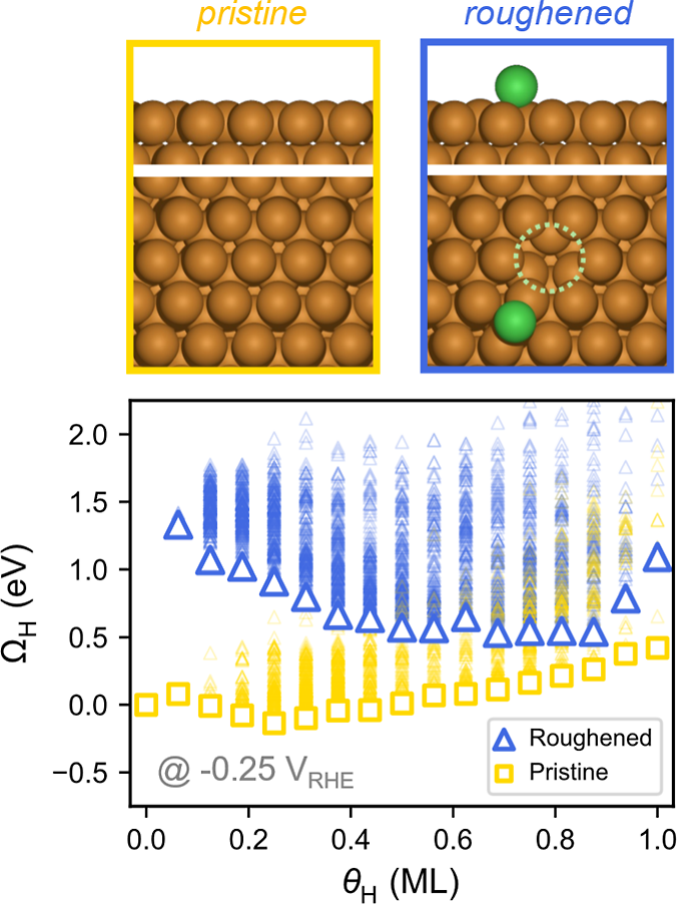
Grand canonical free energetics of pristine and roughened Cu(111) under varying H coverage. Self-roughening with an adatom–vacancy pair is thermodynamically unfavorable at θ_H_ = 0.56 ML and within the full coverage range at HER relevant potentials. Roughened and pristine surface states are plotted as blue triangles and yellow square markers, respectively. Global minimum and local minima of each H coverage are plotted with bold or semitransparent colorings, respectively. Structures of the pristine and roughened surface Cu arrangement are shown in the upper panel with atomic color code: pristine Cu – brown, adatom Cu – green, vacancy – dotted circle.

Previously the impact of Cu(111) single crystal preparation by different means, ranging from electropolishing, to sputter–annealing cycles in ultrahigh vacuum, or induction annealing in a protective atmosphere, has been evaluated by voltammetry. The lower temperature electropolishing process in combination with surface oxidation that occurs during transfer to the EC-MS cell usually results in a rougher interface with narrower terrace widths (and thereby a higher number of defects and step edges) as quantified in the literature by the ratio of two OH^–^ adsorption waves.^51^ In a related fashion, voltammetric cycling in the presence of OH^–^ is associated with the continuous evolution of the electrode structure, the magnitude of which is sensitive to the negative potential vertex.^52–54^ For the present purposes, the impact of H_ads_ coverage on the thermodynamics of Cu(111) roughening was investigated by grand canonical free energetics within the ensembles from constrained GCGA minima searches (Figure S18). As summarized in Figure 2, the pristine Cu(111) is thermodynamically more stable than a surface with an adatom–vacancy pair across the whole H_ads_ coverage range relevant to the HER (Figure S19). Thus, H_ads_ by itself should stabilize the surface against such roughening perturbations, consistent with the smooth (4 × 4) adlayer structure reported for Cu(111) in ECSTM studies.^11,12^

The impact of O_2_ on the Cu surface and hydride formation was then examined by introducing a 50% mix of O_2_ in He into the carrier gas of the EC-MS cell (Figure 3a) at a flow rate of 1 mL min^–1^ at atmospheric pressure. The gas mixture dissolves into the ≈120 μm electrolyte layer (Figure S4) according to Henry’s Law. The ORR is already evident at 0.2 V and increases rapidly in rate until it becomes mass transport limited below 0.0 V at a current density of −450 μA/cm^2^ as shown in Figure 3b.

**Figure 3:**
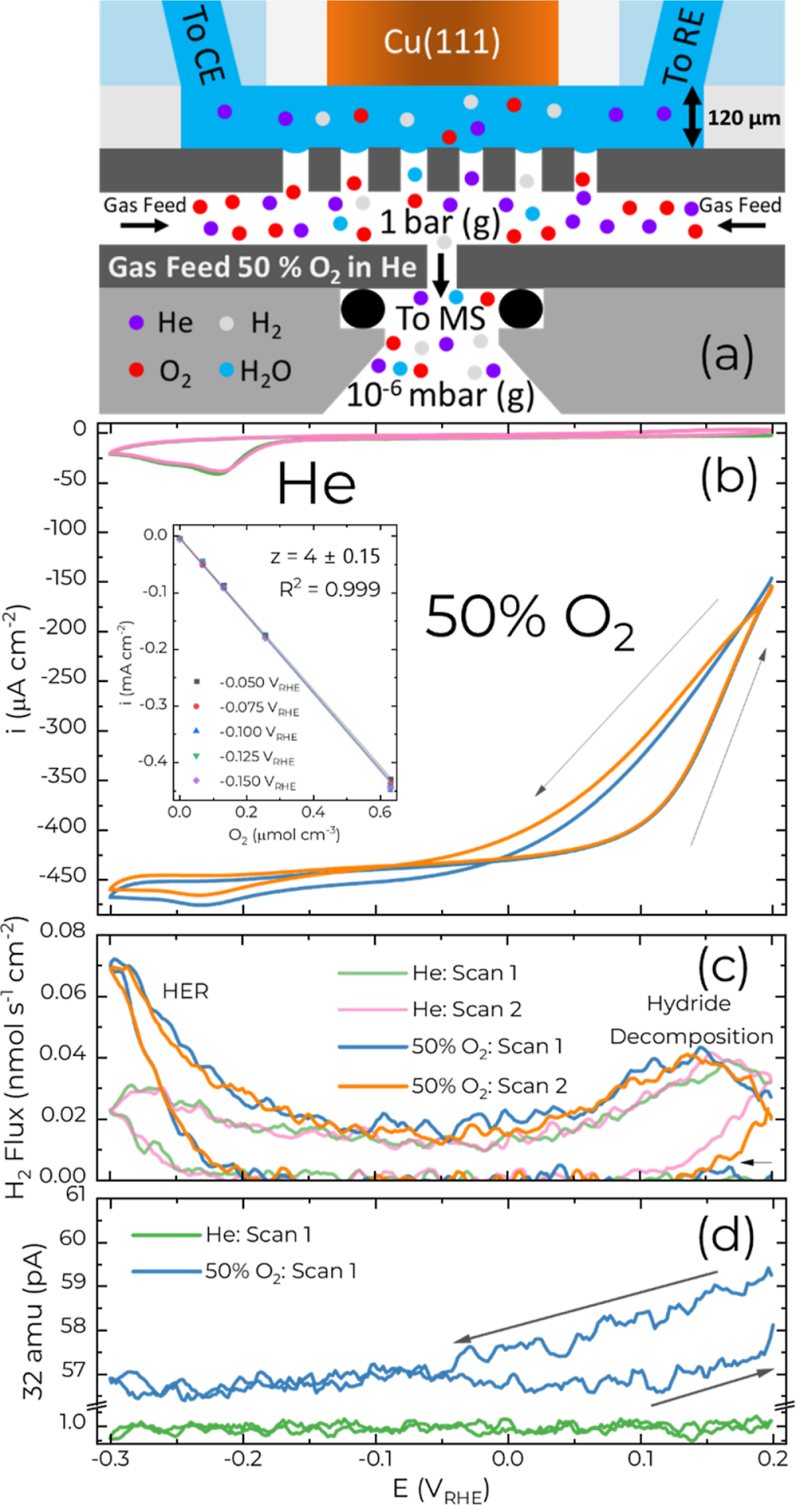
(a) Schematic of the EC-MS with 50% O_2_ in He as the carrier gas. CE and RE stand for counter electrode and reference electrode, respectively. The electrolyte thickness ≈120 ± 10 μm was determined using impulse measurements (Figure S4) (b) Cyclic voltammetry at 10 mV s^1^ on Cu(111) in 0.1 mol L^–1^ HClO_4_ in either He or 50% O_2_ in He. The inset shows current densities (and standard deviation) averaged over 60 s at potentials between −0.1 V and −0.15 V, within the ORR mass transport limited region. (c,d) Corresponding (c) H_2_ flux and (d) 32 amu measured during the cyclic voltammetry. The arrow in (c) shows the start of the first scans with He and 50% O_2_ as the carrier gas. The curves in (c,d) were smoothed using 5 point adjacent-averaging.

The coplanar geometry of the cell enables the comparison of the measured limiting current density (*i*, mA cm^–2^) with that calculated from the solubility of O_2_ (Csat_O_2__, 1.22 mmol L^–1^), its diffusion coefficient (*D*, 1.93 × 10^–5^ cm^2^ s^–1^) and the EC-MS electrolyte thickness of (δ, 120 ± 10) μm, as demonstrated using Fick’s law, eq 1.^55^

1 $$i=zFD\frac{{Csat}_{O_{2}}}{\delta }$$

The measurement of the current density averaged over the last 60 s from pulse measurements (discussed below) between −0.05 V and −0.150 V (Figure 3b inset) gives rise to a monotonic increase in the current density with the O_2_ concentration and a slope of (−637 ± 9) A cm mol^–1^ consistent with 4e^–^ reduction of O_2_ to water (*z* = 4.0 ± 0.15) and RRDE measurements in this potential range.^24^

As the potential is swept to more negative values, the small peak at −0.23 V superimposed on the diffusion limited ORR (Figure 3b) marks the formation of the hydride. The −25 μA/cm^2^ difference between the peak current ≈ −475 μA/cm^2^ and the transport limited ORR value ≈ −450 μA/cm^2^ is close to the peak current observed for hydride formation in the absence of O_2_ (Figure 1). In the first approximation, the process of hydride formation and ORR might appear as a simple linear combination. A closer inspection reveals the single peak is shifted by −15 mV compared to that in the absence of O_2_. The observed shift in formation potential is unlikely to be due to *iR* drop as previously reported impedance measurements using the same cell configuration determined a solution resistance of ≈21 Ohm.^23^ The additional current from ORR ≈85 μA (≈430 μA cm^-2^) would only lead to a potential drop of ≈2 mV.

Surprisingly, the H_2_ flux measurement reveals that the presence of O_2_ leads to an unanticipated 3-fold increase in the HER rate at the negative vertex. On the return sweep, hydride decomposition occurs near 0.15 V by H_ads_ recombination to H_2_, Figure 3c. This overlaps with the transition to the mixed kinetic control of the ORR, which is delayed on the return sweep as reflected in the hysteresis near 0.1 V, suggesting that the hydride surface is more catalytic for ORR compared to the hydride free surface. It is less clear if H_ads_ is a reactant in the ORR. Assuming the hydride formed at negative potentials corresponds to the same coverage in the presence or absence of O_2_, then a similar quantity of H_2_ collected upon hydride decomposition indicates H_ads_ is not consumed in the ORR. The EC-MS shows a modest decrease in the 32 amu signal (≈4%) in the membrane channel due to the draw down associated with the mass transport limited ORR at negative potentials on the Cu(111) electrode (Figure 3d). Likewise, the 32 amu signal also follows the hysteretic behavior exhibited by voltammetry. Further complexity to understanding the hysteresis near 0.1 V in Figure 3b,d is provided by rotating ring disc experiments of ORR on Cu(111) in 0.1 mol L^–1^ HClO_4_ where a change in selectivity toward H_2_O_2_ versus H_2_O production occurs.^24,27^ For Cu electrodes, hydrogen peroxide production is observed at potentials where the equilibrium Cu^+^ activity increases and cross reaction with O_2_ occurs. Indeed, studies of Cu under, and near, open circuit conditions indicate that the Cu^+^ intermediate in Rxn 2 and 3 is so effective in scavenging O_2_ to yield H_2_O_2_, via Rxn 4 and 5, that O_2_ never reaches the electrode surface.^26,56–59^

2 $$Cu\to {Cu}^{+}+e^{-}$$

3 $${Cu}^{+}\to {Cu}^{2+}+e^{-}$$

4 $$O_{2}+H^{+}+e^{-}\to {HOO}^{*}$$

5 $${HOO}^{*}+H^{+}+e^{-}\to H_{2}O_{2}$$

As the potential is advanced to more negative values, the Cu^+^ activity adjacent to the interface decreases and O_2_ is able to reach the Cu electrode where 4 e^–^ reduction to water proceeds. Additionally, the present work reveals that as the potential is decreased below −0.2 V, the interaction between O_2_ and the hydride surface leads to a surprising increase in the HER reaction as measured directly by EC-MS (Figure 3c).

Voltammetry at different scan rates reveals similar characteristics with the ORR under mass transport control with t^–1/2^ relaxation of the boundary layer thickness reaching the EC-MS cell width, and thereby steady-state conditions for scan rates ≤20 mV s^–1^. In the first approximation, the increases in polarization and magnitude of the hydride formation peak both scale with the scan rate, Figure S7. Further cross talk between ORR, HER, and the hydride phase is examined by altering the negative vertex potential during voltammetry in 50% O_2_ (Figure 4). The maximum hydride coverage increases while while decomposition is slightly accelerated when the vertex potential is shifted more negative, from −0.300 V to −0.375 V, where the peak hydride decomposition H_2_ flux shifts from 130 to 75 mV, respectively (Figure 4b). This is similar to observations previously made in O_2_ free 0.1 mol L^–1^ HClO_4_.^23^ Significantly, the negative shift in the hysteretic transition from mass transported limited ORR kinetics to mixed control also overlaps with the shift in the peak hydride decomposition and tends to rule out the presence of significant halide contamination­(Figure 4a).

**Figure 4:**
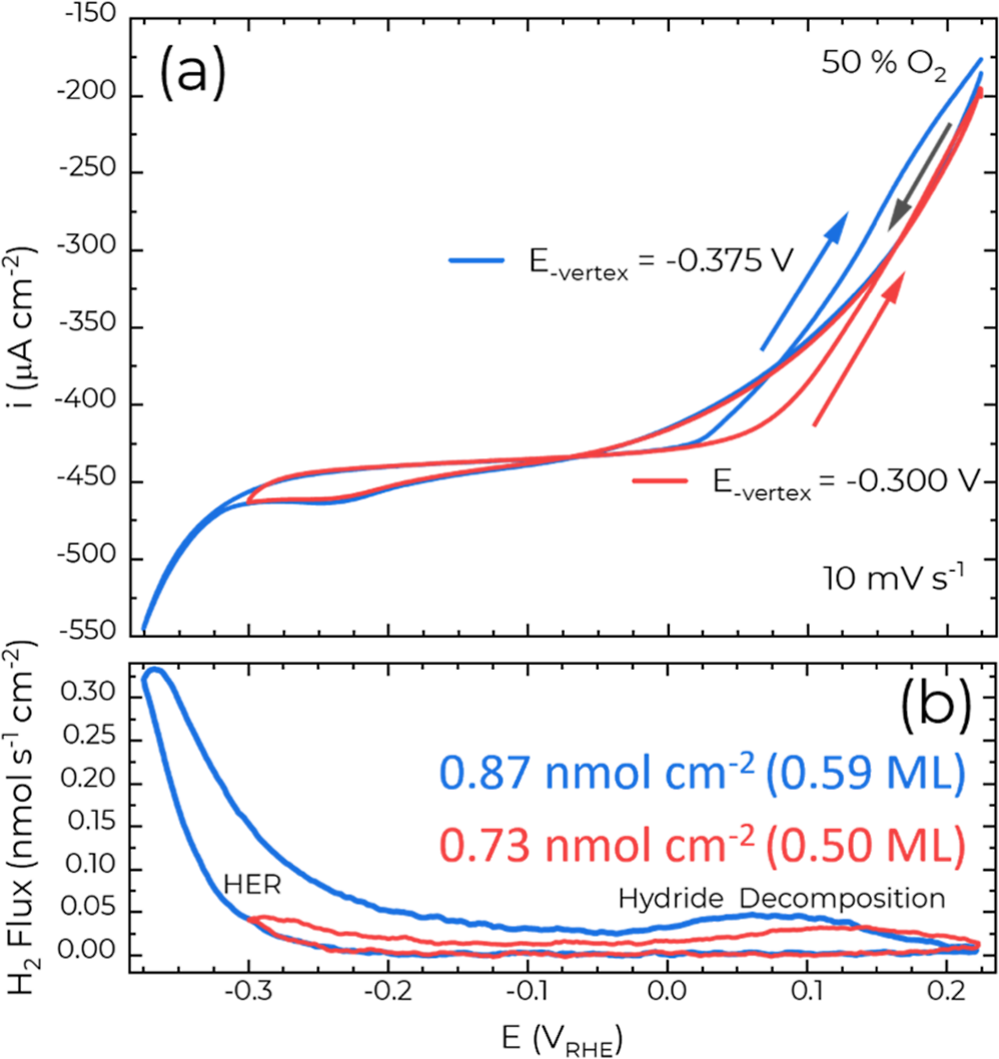
ORR kinetics, hydride coverage (and decomposition) as a function of negative vertex potential during voltammetric EC-MS on Cu(111) in 0.1 mol L^–1^ HClO_4_ with 50% O_2_ as the carrier gas.

### Potential Pulse Measurements

Potential pulse EC-MS measurements were used to better understand the relationship between hydride coverage, HER kinetics, and O_2_ in 0.1 mol L^–1^ HClO_4_.^14,23^ In this experiment, the potential was stepped between a rest state, *E*_Rest_ = 0.175 V, and progressively more negative potentials, *E*_Pulse_, in −25 mV increments for 2 min intervals (Figures 5, S8) with the intent to disentangle the transient processes from steady-state processes and the reversible from longer-lived structural changes.

**Figure 5:**
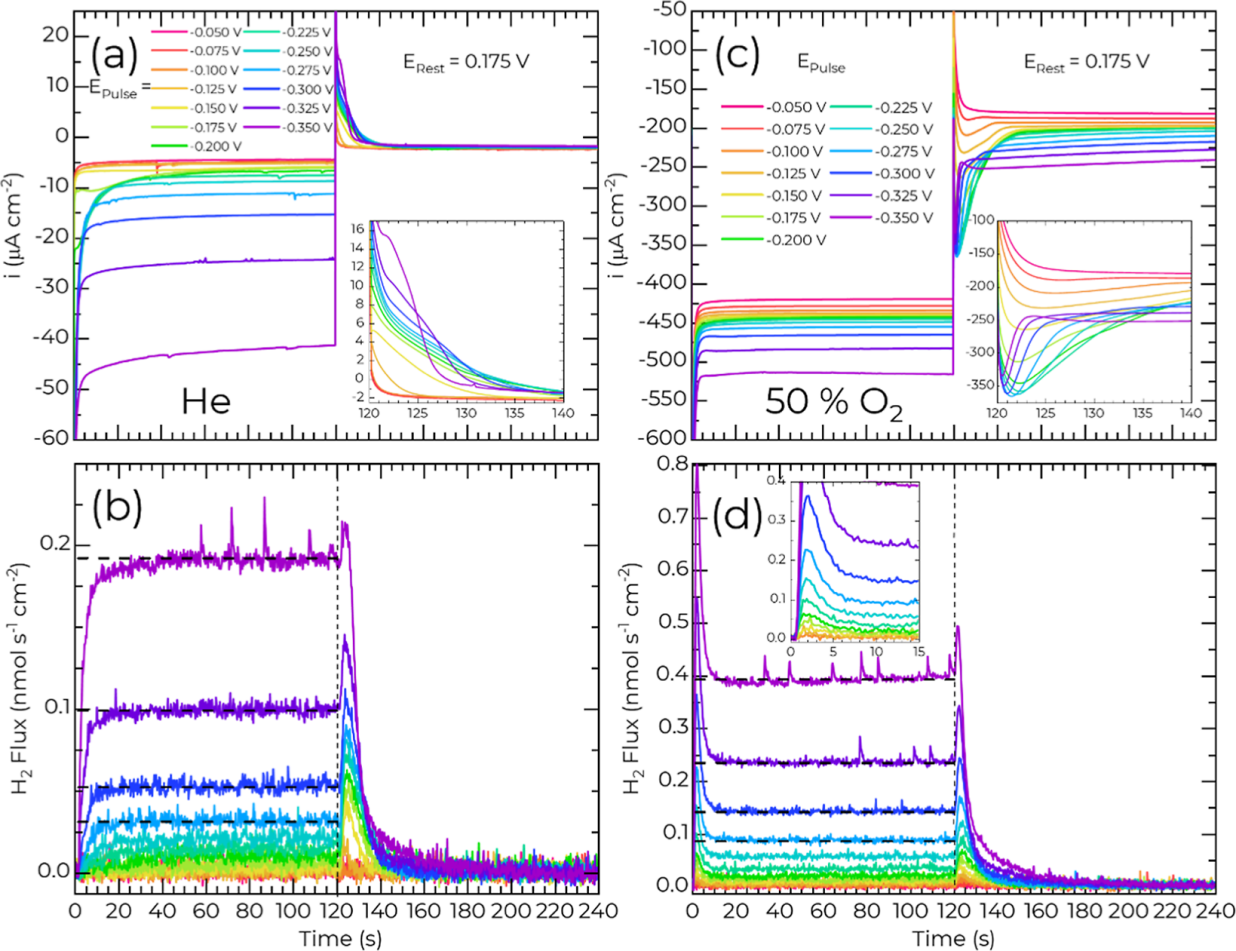
(a,c) Chronoamperometry and (b,d) H_2_ flux from potential pulse measurements on Cu(111) in (a,b) He saturated or (c,d) 50% O_2_ saturated 0.1 mol L^–1^ HClO_4_. The horizontal dashed lines in (b,d) H_2_ flux plots indicate the steady-state HER flux at the four most negative *E*_Pulse_ determined by averaging the last 60 s of the flux during *E*_Pulse_. The vertical dashed line indicates the transition to *E*_rest_. The insets in (a,c) zoom in on the transient periods of *E*_Rest_. The inset in (d) zooms in on the transient period at *E*_Pulse_.

Prior to −0.15 V only trace ORR, due to picomolar O_2_ concentrations, is observed.^14^ Hydride is first observed at −0.15 V (Figure S10a), where the current density increases from −4 to −7 μA cm^–2^ (Figure S9a). At incrementally more negative potentials, the evolution of the hydride phase with potential is monitored via the current passed within the first ≈30 s (Figures 5a and S9a). At −0.175 V, the current peaks near 10 s at −10 μA cm^–2^ before falling off to a nearly identical steady-state current as −0.15 V. Stepping to −0.25 V increases the magnitude and decay rate of the transient current associated with the 2D nucleation and growth of the surface hydride phase that is further accelerated at larger overpotentials and superimposed with proton reduction to H_2_ and double layer charging.

Returning the electrode to *E*_Rest_ following hydride formation produces an oxidative transient (Figure 5a inset) with a magnitude and duration that strongly depends on the preceding *E*_Pulse_. This transient represents a combination of double layer charging and hydride oxidation to proton. *Pro memoria ,*in the case of sulfate or halide electrolytes, anion adsorption on Cu contributes additional oxidative charge.^23,60^ A time constant inversion occurs at *E*_Rest_ once *E*_Pulse_ < −0.3 V with the transient current at *E*_Rest_ decreasing to a steady state in 8 s following polarization at *E*_Pulse_ of −0.35 V. The inversion suggests that polarization at sufficiently negative potentials induces surface restructuring that alters the kinetics of the subsequent hydride decomposition process in a manner consistent with the creation of new surface sites that were not present on the pristine Cu(111) surface.

When the potential is stepped back to *E*_Rest_ (from ≤ −0.125 V), the H_2_ flux from the EC-MS (Figure 5b) provides a clear indication of hydride formation by tracking its subsequent decomposition via recombination to H_2_ (H_2_,_hydride_). The H_2,hydride_ peak continues to grow as *E*_Pulse_ is stepped more negative to approach a subtle plateau suggestive of saturation by −0.25 V. Hydride formation is superimposed on comparatively modest increases in the steady-state HER (Figures 5b and S11) over the same potential range. For *E*_Pulse_ < −0.25 V, the increase in HER further complicates the analysis of the subsequent hydride decomposition at *E*_Rest_ due to an overlap between the decaying HER signal associated with the diffusion limited capture of the so-generated H_2_ and the H_2_ produced by hydride decomposition.

With the addition of 50% O_2_ to the EC-MS carrier gas, the current response during potential pulses is dominated by the ORR at both *E*_Pulse_ and *E*_Rest_. Consistent with voltammetry (Figure 3b), the steady-state current during *E*_Pulse_ exhibits mass transported constrained ORR (Figure 5c), only changing from ≈ −420 μA cm^–2^ to ≈ −470 μA cm^–2^ from −0.05 V to −0.30 V. The increase over this range convolves the contributions from proton reduction (≈−50 μA cm^–2^) and hydride formation superimposed on the larger ORR current (Figure S9b).

At *E*_Rest_, the electrode exhibits mixed control for the ORR reaction with the steady-state current density (≈−200 μA cm^–2^) just under half that of the transport limited value (−450 μA cm^–2^) seen by voltammetry in Figure 3b. Notably, this steady-state current depends systematically on the preceding *E*_Pulse_. As *E*_Pulse_ goes from −0.05 V to −0.3 V, the magnitude increases from −180 μA cm^–2^ to −240 μA cm^–2^ with minor decay occurring over the 2 min hold. Likewise, the transient peak current at *E*_Rest_ increases in magnitude from +180 μA cm^–2^ to −350 μA cm^–2^ as *E*_Pulse_ decreases from −0.05 V to −0.225 V, saturating for *E*_Pulse_ < −0.225 V. However, the duration of the transient exhibits a bimodal dependence on the preceding *E*_Pulse_. Initially, from *E*_Pulse_ of −0.075 V to −0.225 V, the duration of the transient at *E*_Rest_ extends from ≈10 to ≈30 s. As *E*_Pulse_ is stepped ≤0.225 V, the duration of the transient decays to about 3 s by −0.35 V reflecting a more rapid deactivation of the ORR kinetics due to a change in reactivity of Cu associated with the surface hydride. This rapid current fall off is a direct indication that up to 0.75 ML coverage, the hydride phase accelerates the charge-transfer kinetics of the ORR reaction relative to the hydride-free surface (i.e., decomposition of the surface hydride (Figure 5d) leads to the decay of the ORR reaction at *E*_Rest_ evidenced by the transient). Additionally, the elevated steady-state ORR activity (e.g., −240 μA cm^–2^ vs −180 μA cm^–2^) reveals the existence of nonhydride, reconstructed, surface sites that are more active for ORR than the original Cu surface.

A comparison of the mass spectrometer H_2_ flux measurements also reveals important differences specific to HER, hydride formation, and decomposition that are induced by ORR on Cu(111) (Figure 5d). The transport limited nature of the ORR prevents a direct evaluation of the impact of the hydride on the ORR reaction kinetics. Superposition of hydride formation with the transport-limited ORR, evident in the voltammetry (Figure 3) and chronoamperometry (Figure 5c), might suggest a linear combination of two reactions. However, closer inspection reveals multiple features that speak to more complicated interactions. In the presence of O_2_, the initial transient response is marked by a very sharp spike in transient H_2_ production that mirrors the current transient (Figure 5d inset and Figure S10b) contrasting with the asymptotic rise (as hydride forms) to steady-state H_2_ production seen in the absence of O_2_ (Figure 5b). For *E*_Pulse_ ≤ −0.175 V, both the transient and steady-state HER rate measured by the mass spectrometer is notably larger than observed in the absence of O_2_ (Figure 5, summarized in Figure S11). For example, at −0.35 V, the steady-state rate of H_2_ generation is 400 pmol s^–1^ cm^2^ compared to 190 pmol s^–1^ cm^2^ in the absence of O_2_. The result aligns with differences in the voltammetry shown in Figure 3c.Figure S1 Ultimately, this suggests that with ORR present, stepping down to hydride formation generates new active sites that catalyze the HER. Interestingly, the potential and reactant-driven surface rearrangements are analogous to those reported for other reduction reactions on Cu surfaces such as CO_2_ or NO_3_^2–^ reduction.^1,18,61,62^

An alternative explanation of the initial large ORR current at *E*_Rest_ and its subsequent accelerated decay at *E*_Pulse_ < −0.2 V might be the transient consumption of H_ads_ by ORR. This would lead to a decrease in the amount of H_ads_ available for recombination unless the amount of H_ads_ corresponds only to the portion of H_ads_ that is otherwise oxidized back to proton in the absence of O_2_. Accordingly, attention is given to determining the relative contributions of these two processes by the integral analysis of the current and H_2_ flux transients.

### Quantitative Analysis of Hydride Coverage

In the absence of O_2_, hydride coverage can be quantified using a steady-state approximation detailed in prior publications.^14,23^ This involves separation of transient charges (*q*_P,Transient_, *q*_R,Transient_) and steady-state charges (*q*_P,SS_, *q*_R,SS_) from the total charge (*q*_P,Total_, *q*_R,Total_) for both *E*_Pulse_ and *E*_Rest_ (Figure S12). The respective steady-state charges are calculated, under the assumption that the rates of Faradaic reactions are constant, taking the average of the current over the final 60 s of *E*_Pulse_ or *E*_Rest_, and multiplying by the total pulse or rest time (120 s each). The subtraction of the steady-state charge from the total charge during *E*_Pulse_ or *E*_Rest_ yields the transient charge, *q*_P,Transient_ or *q*_R,Transient_, respectively. Initially, before hydride formation, the transient charges for *E*_Pulse_ and *E*_Rest_ are approximately equal with opposite polarity, indicative of simple capacitive charging of the interface (Figure S12b). The onset of hydride formation introduces asymmetry in the net transient charges due to nonfaradaic recombination at *E*_Rest_. Specifically, *q*_P,Transient_/*q*_R,Transient_ increases from around 1 at *E*_Pulse_ = −0.125 V (prehydride formation) to about 2.4 at −0.25 V, where the surface hydride coverage reaches a plateau. The corresponding *q*_P,Transient_ and q_R,Transient_ are (−226 ± 21) μC cm^–2^ and (+87 ± 6) μC cm^–2^ by taking the difference in the minimum charge (*q*_min_) and charge at the plateau (Figure S12b), respectively. The former corresponds to a coverage of (0.80 ± 0.08) ML of H_ads_. Taking the oxidative charge in combination with the EC-MS measurements enables partitioning hydride decomposition between oxidation and H-recombination (H_2,Hydride_). Accordingly, the (+87 ± 6) μC cm^–2^ oxidative charge is assigned to H_ads_ conversion to hydronium (0.31 ± 0.03) ML H_ads_, while the other ≈ 0.5 ML H_ads_ follows the recombination pathway as sensed by EC-MS.

In the above analysis, and prior work, deconvolving the H_2_ signal leveraged the total collection capacity of the EC-MS thin-layer cell platform^28^ combined with the steady-state approximation for HER such that the EC-MS detection delay is due solely to H_2_ diffusion across the thin layer of electrolyte.^14^ However, when significant O_2_ is introduced into the cell via the carrier gas, the unanticipated sharp transient increase in H_2_ flux observed upon stepping to *E*_Pulse_ in Figure 5d indicates that the second condition is no longer valid. Upon stepping back to *E*_Rest_, a more rigorous strategy is needed to account for the collection of the remaining H_2_ that was generated during the HER at *E*_Pulse_. Namely, the time evolution for the collection of the H_2_ flux across the electrolyte layer (Figure S13a) is evaluated.^28^ For validation, the analysis is applied to the nominally O_2_-free case, where agreement with prior steady-state analysis is anticipated. Preliminary work with a single time constant for H_2_ collection overestimates the rate of decay and undervalues the amount of H_2_ collected, thereby overestimating the amount of hydride formed.^28^ Adopting a second time constant in the fitting equation was shown to be helpful in previously published potential pulse measurements on Ag (Figure S13b).^14^ In the two-component time constant model, the first term corresponds to H_2_ diffusion between the electrode and EC-MS chip, while the second term describes the slower collection of H_2_ captured from recessed volumes associated with the various cell ports. The ratio of the pre-exponential factors (*A*_1_, *A*_2_) and the second decay constant (*t*_2_) were held constant (at the best fit values obtained with the silver electrode) for fitting the *E*_Rest_ decay related to collecting the H_2_ remaining from the *E*_Pulse_ HER cycle (Figure S14). Subtracting this result from the measured transient leaves the remaining flux increment attributable to hydride decomposition, H_2,hydride_ (Figure S14c). Comparing H_2,Hydride_ determined using both fitting approaches (Figure S14d) highlights the impact of the second decay term particularly in accounting for the increased H_2_ generated by the HER at a more negative *E*_Pulse_.

Determination of H_2,Hydride_ in the absence of O_2_ yields good agreement between the transient analysis (using the exponential decay) and steady-state analysis (the total collection method) with the results falling within the standard deviation of the respective measurements (Figure S15). Figure 5b. Because hydride accumulates throughout the pulse and decomposes rapidly, EC-MS more clearly resolves the onset of hydride formation than chronoamperometry (evident at E^_Pulse_^ = -0.125 V). Nevertheless, the monotonic relationship between hydride coverage and *E*_Pulse_ is evident from both methods within the range of −0.15 V to −0.225 V. A plateau is reached between −0.225 V and −0.275 V that corresponds to a hydride coverage of (≈0.53 ± 0.05) ML, when both evaluation techniques are averaged. It is noteworthy that this is lower than the 0.67 ML observed in 0.1 mol L^–1^ H_2_SO_4_ using the same procedures.^14^ This is ascribed to sulfate adsorption stimulating recombination at the expense of the oxidative desorption pathway. It is plausible that below −0.3 V additional surface or subsurface hydride may form; however, the challenge of measuring increments of submonolayer quantities of H_ads_ (e.g., H_2,hydride_ or *q*_P,Transient_) in the presence of ever increasing H_2,HER_ quantities (or *q*_P,SS_, in the case of charge) introduces increasing error margins in the measurement as evident in Figure 6 (and Figure S15). Recent computational studies of H adsorption on Cu(111) explored the potential-dependent energy and coverage of H_ads_.^11^ The potential domains of stability for different coverages and associated adlayer structures were described. Of particular interest are the hydride phases between 0.56 and 0.75 ML H_ads_ with periodic (4 × 4) unit cell structures. For *E* < −0.360 V (pH 1), theory predicts surface reconstruction involving organized adatoms and vacancy structures. Indeed, the increase in coverage below ≈ −0.325 V in Figure 6 may reflect the impact of such reconstruction.

**Figure 6:**
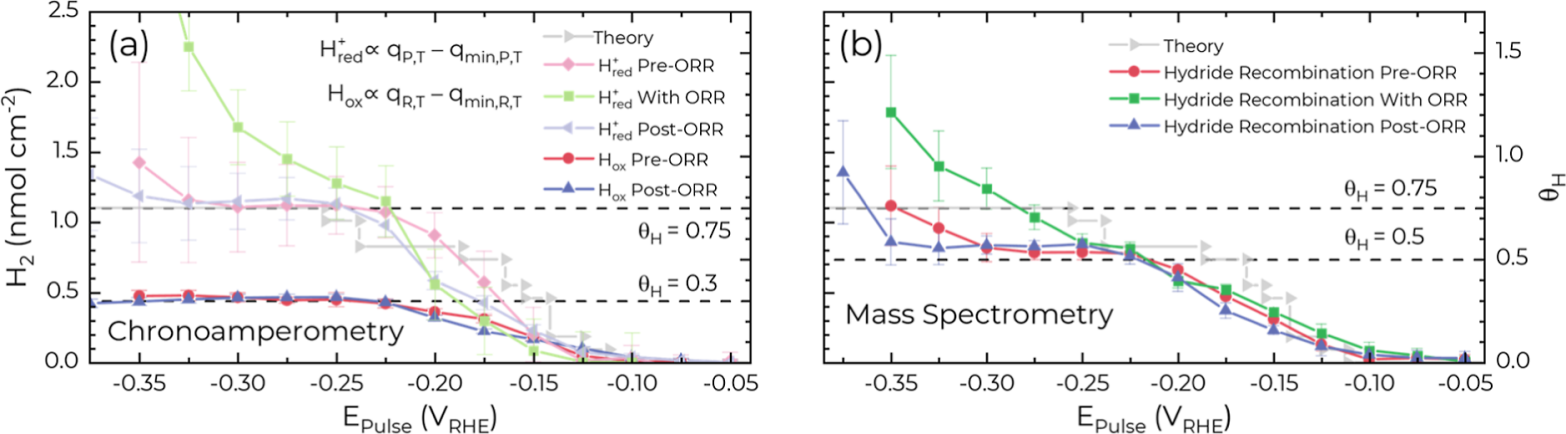
Hydride coverage determined from (a) chronoamperometry (Figures 5a,b, S12, and S17) and (b) H_2_ flux from the MS (Figures 5c,d, and S17b) during potential pulse measurements compared to the coverage (denoted “theory”) predicted by computational simulations from Cheng et al. for pH 1.^11^ Coverage from the MS was determined using a two-term exponential fit of the decaying HER signal. Adapted from ref. [11] (Cheng et al., Angewandte Chemie International Edition 2023, 62, e202218575). Copyright © 2023 Wiley-VCH GmbH. Reproduced with permission.

In the presence of O_2_, evaluating hydride coverage using chronoamperometry is challenging due to significant contributions from the ORR. Additionally, the current transient observed upon returning to *E*_Rest_ (Figure 5c) exhibits intriguing complexity, as previously detailed. Conversely, the H2 flux is more straightforward to interpret after calibration with the appropriate level of O_2_ present (see the Supporting Information).

Upon stepping to *E*_Pulse_ = −0.150 V, a prominent transient spike in the H_2_ flux necessitates analysis using a two-term exponential decay for interpreting the hydride-related transient upon returning to *E*_Rest_. With 50% O_2_ present, hydride formation onset remains evident at potentials below −0.100 V, and the evolution of H_ads_ coverage is similar to conditions without O_2_ down to −0.225 V (Figure 6b). At more negative potentials, however, the presence of the ORR causes H_ads_ coverage to continue increasing monotonically, surpassing 1 ML for potentials below −0.325 V. Although a slight inflection near 0.5 ML is noticeable, the clear plateau observed under the O_2_-free conditions is absent. The increase in H_2_ hydride generation in the presence of O_2_ and ORR suggests that the ORR intermediates modulate the hydrogen binding energies and/or lead to the formation of new surface sites.

### Simulations of Active ORR Intermediates and Hydride on Cu(111)

Given the evidence that ORR intermediates restructure catalytic surfaces, how these species interact with the hydride-covered Cu(111) surface is explored using DFT and MD simulations. Here, *OOH and *OH are considered as the primary ORR intermediates at the dilute coverage limit and their most stable adsorption configurations are sampled on metallic or hydride (θ_H_ = 0.75 ML) surfaces (Figures S20–S23).

The thermodynamics for the metallic and hydride surfaces to reconstruct, with or without adsorbed ORR intermediates, are calculated using GC-DFT near the HER onset, -0.25 V RHE, and are summarized in Figure 7a,b. ORR intermediates are found to promote the spontaneous roughening of the hydride surface: both adsorbates stabilize the formed adatom–vacancy pair by binding to the bridge site on the vacancy perimeter, which energetically goes downhill by −0.04 eV (*OOH) and −0.02 eV (*OH) compared to those on the bridge sites of the pristine hydride surface. Note that the surface roughening requires both the surface hydride and the ORR intermediate, as the former weakens surface Cu–Cu bonds, while the latter serves as a strong ligand to thermodynamically stabilize the undercoordinated moieties.^37,63^ Roughening of all other investigated configurations that lack either surface hydride or an ORR intermediate is thermodynamically unfavorable.

**Figure 7:**
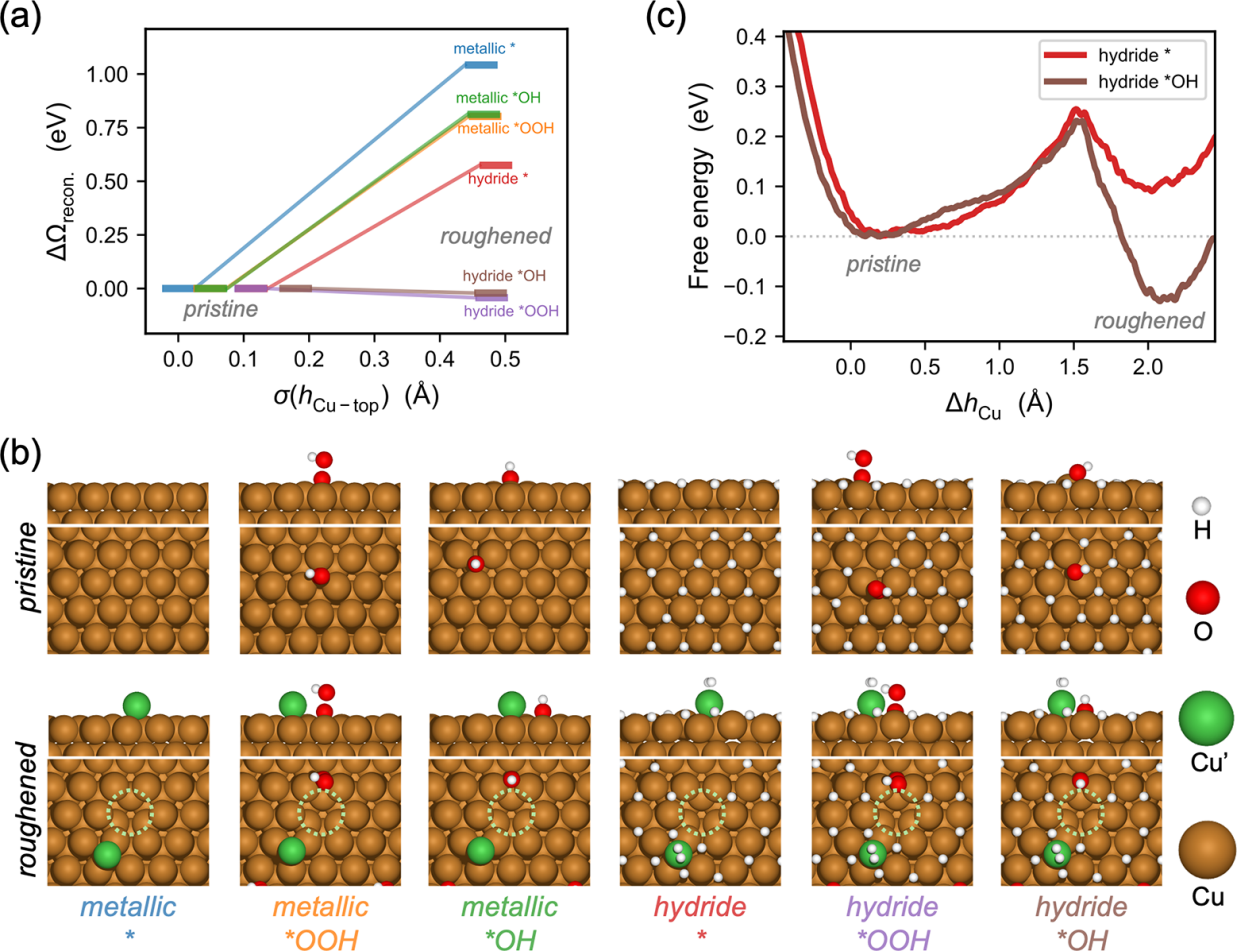
Thermodynamics and kinetics of ORR intermediate-induced roughening of the hydride surface. (a) Grand canonical free energy diagram of Cu(111) reconstruction in metallic or hydride state and with or without adsorbed ORR intermediates at -0.25 V_RHE_, with the roughness of top-surface Cu as the reaction coordinates. (b) Front and top views of all involved structures in the energy diagram with atomic color code: H – white, O – red, pristine Cu – brown, adatom Cu’ – green, vacancy – dotted circle. (c) Free energy profile of pristine-to-roughened transition of the hydride surface with or with *OH, from constrained ab initio molecular dynamics and thermodynamic integration.

To confirm if the roughening process is kinetically viable, we perform constrained AIMD simulations and thermodynamic integration (Figure S24) to compute the free energy profile of the pristine-to-roughened transition of the hydride surface with or without a surface *OH. The barriers for surface roughening via formation of adatom–vacancy pair are accessible, at 0.22 and 0.25 eV for both cases (Figure 7c), suggesting that the surface Cu atoms can rapidly rearrange and form a distribution of accessible hydride states (rich local minima in Figure 2), i.e., exhibit fluxional behaviors. Note that, although “fluxionality” is often associated with clusters, growing theoretical and experimental evidence shows that extended surfaces, especially low-cohesive-energy metals under heavy adsorbate coverage,^64–67^ can also access dynamic ensembles of coexisting metastable configurations within catalytic time scales.^68^ Therefore, the Cu system studied herein should not be kinetically constrained and can follow the thermodynamics to roughen in the presence of both surface hydride and ORR intermediates. Consequently, the roughened surface would be able to host more H_ads_ than the pristine surface (Figure 2), which rationalizes the higher maximum H coverage in the presence of 50% O_2_ (Figure 6b). Furthermore, the Cu adatom has previously been shown to be a highly active HER site and likely contributes to the enhanced HER activity observed under ORR conditions.^11^

The fluxionality of the hydride surface prompts further analysis of the coupling between key ORR steps and the surface roughening process. Here, we focus on *OOH dissociation, which involves O–O cleavage. As shown in Figure S25a, on a metallic surface, the *OOH prefers to dissociate on the top surface and produce *O and *OH that occupy the hollow sites, which we denote as the top-surface pathway. However, on the hydride surface, the *OOH prefers to “dig” into the surface while it dissociates, producing a subsurface *O and a top-surface *OH (Figure S25b), which we denote the subsurface pathway. On the hydride surface, the subsurface pathway is more thermodynamically favorable than the top-surface pathway by 0.43 eV at the HER onset. Moreover, the formation of the subsurface *O causes strong local buckling of the Cu atoms above it, which, under the coinfluence of the top-surface *OH, elevates the adjacent Cu atoms into a distinct raised atom state. This behavior echoes previous calculations where at high overpotentials H-stabilized adatoms form on Cu(111) and serve as active HER sites. Due to the subsurface trapping of *O, the raised atoms are expected to have a longer lifetime than the above-discussed adatom–vacancy pairs and could be responsible for the irreversible character observed after cycling in higher concentrations of O_2_ (vide infra).

Here, the simulations indicate that ORR intermediates can drive similar restructuring even at lower overpotentials, effectively generating reversible and irreversible HER-active sites earlier in the potential window. This mechanistic link between oxygen species and hydrogen catalysis provides support for the ORR-stimulated HER enhancement observed by EC-MS.

### Dependence of HER with Varying Oxygen Concentration

The relationship between the O_2_ and HER kinetics was further investigated using potential pulse measurements while varying the O_2_ concentration in the carrier gas (5, 10, 20, and 50%), followed by re-evaluation in the absence of O_2_. The reaction intermediate coverage is expected to scale with O_2_ concentration and the related increase in adatom population accounts for the acceleration of the HER. The H_2_ flux from HER is tracked in the presence of different O_2_ concentrations for *E*_Pulse_ = −0.3 V in Figure 8a. As the O_2_ level is increased from 0 to 10%, there is a monotonic increase in the steady-state rate of HER with a further increase for 50% O_2_. For O_2_ levels > 5%, the initial transient H_2_ flux is enhanced to an even greater degree. The response demonstrates a link between the magnitude of the O_2_ reduction and its activation of the HER. Upon returning to a nominally O_2_-free condition, the HER activity is diminished slightly relative to the initial condition. In contrast to the HER, upon stepping to *E*_Rest_, the H_2,Hydride_ decomposition peak appears mostly unchanged. This points to a primary correlation of the HER to ORR rather than the hydride surface coverage per se. Repeating the experiment at different *E*_Pulse_ values (Figure 8b) reveals that the most pronounced increase in steady-state HER activity occurs between 0 and 10% O_2_ concentrations; thereafter, the steady-state HER rate at *E*_Pulse_ ≥ −0.275 V either plateaus or decreases relative to 10% O_2_. However, for *E*_Pulse_ < −0.275 V, the HER activity increases as the O_2_ level is increased from 10 to 50% O_2_.

**Figure 8:**
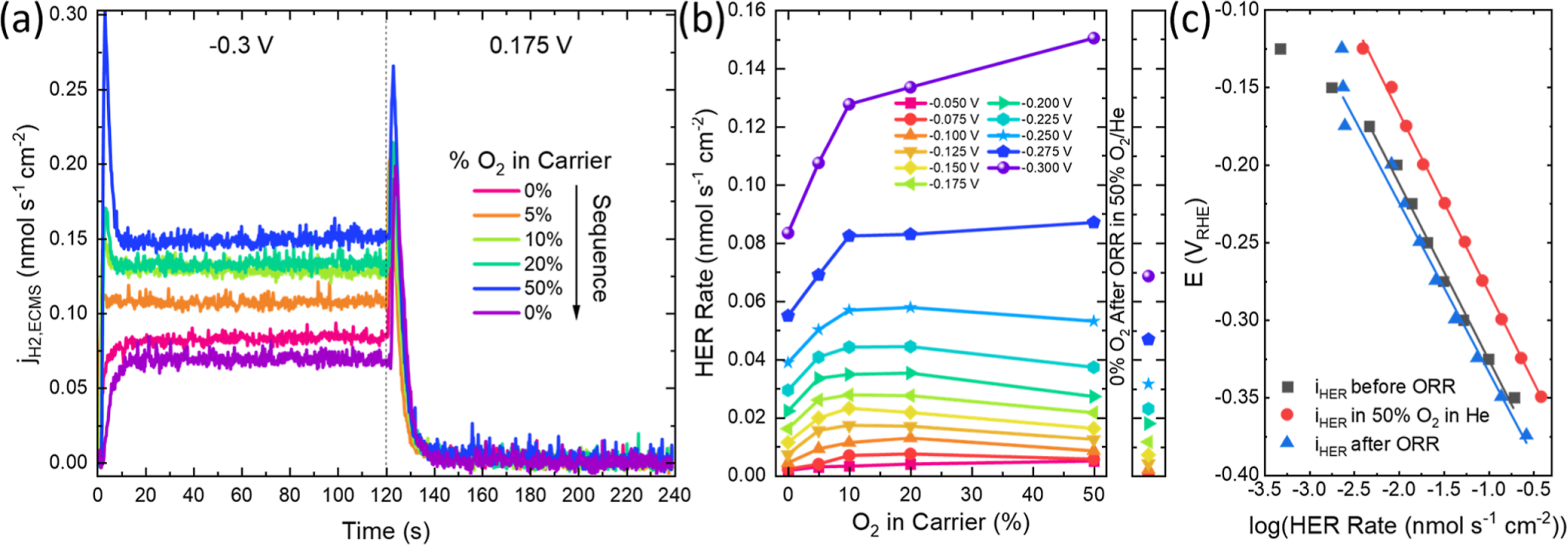
Analysis of ORR influence on HER. (a) Potential pulse with different O_2_ saturations at −0.3 V, (b) steady-state HER rate, as determined by the EC-MS vs O_2_ saturation at all potentials. (c) Log plot of steady-state HER rate before, with 50% O_2_, and after O_2_ exposure. *R* ^2^ for the linear fits are 0.992, 0.998, and 0.992 for before, during, and after 50% O_2_ exposure, respectively.

Following the O_2_ concentration series the steady-state HER rate at 0% O_2_ was remeasured and found to be slightly diminished compared to the pre-O_2_ exposure activity. This was observed to be true for all potentials investigated. The steady-state response is summarized in Tafel plots of the potential activated HER before, during, and after 50% O_2_ exposure (derived from Figures 5 and S17). All three conditions exhibit similar Tafel slopes close to 120 mV dec^–1^ with the marked increase in the HER exchange current density for measurements in the presence of O_2_ as shown in Figure 8c. Summarizing the presence of O_2_ and the ORR leads to an earlier increase in the adatom population that has previously been assigned as catalytic sites for HER.^6^

### Alteration of Cu(111) Following ORR

As with the HER at high overpotentials, the sensitivity of Cu(111) to ORR-induced restructuring and the so-generated vacancies and adatoms will be subject to healing by annihilation or step capture. The dynamics will be potential and time dependent. The impact of such changes on mesoscale structure, hydride formation, and decomposition is assessed by voltammetry. Accordingly, immediately following the ORR measurements, the spectrometer gas feed was switched back to 100% He and fresh Ar purged electrolyte was injected through the thin-layer EC-MS cell. On the first cycle (Figure 9), the initial hydride formation reduction peak at −0.22 V is diminished, while the second peak increases and shifts by −25 mV to −0.275 V, similar to the initial voltammetry presented in Figure 1. On the anodic sweep, the hydride decomposition peak shifts from 0.175 V to the upper vertex of 0.225 V. Extending the negative vertex potential during CVs after 50% O_2_ exposure confirms the continued sensitivity of the hydride decomposition potential to the negative vertex (Figure S16) reported previously.^23^

Potential pulse measurements (Figure S17) in the fresh He-saturated electrolyte reveal a −50 mV shift in potential for hydride formation onset (Figure 6, “Post ORR”). However, this is more evident in the chronoamperometric (Figure 6a) and total collection (Figure S15b) approaches to H_2,Hydride_ determination compared to the two-term exponential decay fit (Figure 6b). Hydride decomposition via recombination still appears to saturate at (≈0.56 ± 0.03) ML, while total cathodic charge predicts saturation at (0.75 ± 0.09) ML between −0.25 V and −0.275 V. Determining whether the H_2,hydride_ coverage increases beyond −0.325 V is challenged by the increasing HER rate and the corresponding increase in statistical measurement error. It is likely that the extended potential pulse sequence results in resetting and/or annealing of surface alteration from prior O_2_ exposure and ORR experiments (i.e., Figure 1). Furthermore, cyclic voltammetry experiments in He (Figure 9a), before and after ORR experiments, unambiguously reveal that ORR alteration of the Cu surface occurs.

**Figure 9:**
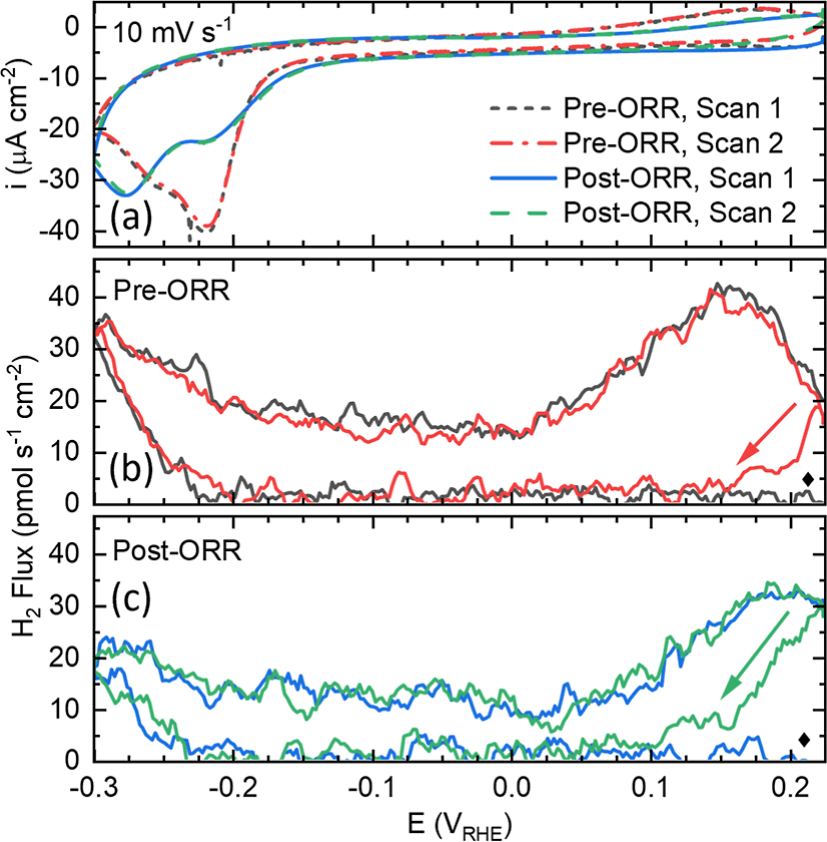
(a) Cyclic voltammogram and (b,c) H_2_ flux comparison from (b) before and (c) after exposure to 50% O_2_ in He. The diamond indicates the start of the first CV for the H_2_ flux plots. While the arrow indicates the start and direction of the 2nd CV.

Additional potential pulse measurements (not shown) were conducted following a similar sequence as in Figure 4 with the O_2_ concentration sequentially increased from 5, to 50% and finally returned to 0%. The resulting hydride-coverage plots (Figures S26), determined by hydride decomposition via recombination, largely mirror the trends in Figure 9, showing the evolution of hydride coverage with potential before and after the exposure to O_2_ exposure. An interesting empirical observation is the enhanced surface hydride coverage at lower overpotentials (≥ 0.2 V) for O_2_ concentrations ≤ 20% (Figure S26a–c). This is congruent with the computational result of the synergistic roughening effect induced by H_ads_ and ORR intermediates on the Cu(111) surface. However, at 50% O_2_ (Figure S26d), this trend reverses, likely due to the more extensive surface reconstruction that impacts hydride formation itself after the exposure to O_2_ exposure. Future investigation will be necessary to fully elucidate this transition.

## Conclusions

The interactions between surface hydride formation, ORR, and HER on Cu(111) in 0.1 mol L^–1^ HClO_4_ were examined by using a combination of electrochemical mass spectrometry and grand-canonical free-energy calculations. Cyclic voltammetry in the Ar-sparged electrolyte reveals two overlapping hydride formation waves that evolve with cycling, reflecting the progressive restructuring of the electrode surface associated with removal of residual oxide species. Polarization to potentials sufficient to generate ≈0.75 ML of surface hydride leads to redistribution of these features, with an increase in the lower overpotential hydride wave accompanied by a corresponding decrease in the higher overpotential wave, while background currents associated with residual oxygen reduction diminish. Grand-canonical free-energy calculations at −0.25 V indicate that the two-dimensional (2D) surface hydride stabilizes Cu(111) terraces against roughening. Thus, in deaerated electrolytes hydride formation and the removal of residual oxo- or hydroxo-species from the surface improve surface quality by reducing the surface roughness.

Introduction of controlled amounts of O_2_ (mixed with the He carrier gas) leads to an increase in hydride formation and a marked increase in HER kinetics at more negative potentials. Simulations show that coadsorbed H with ORR intermediates (OH*/OOH*) drives Cu(111) restructuring both by adatom–vacancy pair generation and subsurface oxygen incorporation. The resulting adatoms enhance HER (supported by Tafel analysis) at negative potentials and provide transient increases in ORR rates at more positive potentials where the reaction is under mixed control (e.g., *E*_Rest_ = 0.175 V). After extended O_2_ exposure, marked redistribution of the two hydride waves is observed, with the more negative potential peak becoming predominant, even more so than when the electrode is initially immersed into the cell. Collectively, these results demonstrate that coupled adsorbates, specifically H_ads_ with ORR intermediates, induce the dynamic restructuring of the Cu surface atoms under electrochemical bias, generating metastable defect motifs that strongly influence catalytic kinetics.

The mechanistic insights reported here extend beyond the specific case of the HER and ORR on Cu(111). Copper-catalyzed reduction reactions such as CO_2_ reduction, nitrate reduction, and NO_*x*_ reduction are all known to involve high surface hydrogen coverages, potential-dependent restructuring, and sensitivity to trace oxidants or oxygenated intermediates. The present findings suggest that hydride-stabilized terraces, as well as oxidant-induced adatom and defect formation, may play a central role in establishing the active surface state of Cu under reducing conditions relevant to these reactions. More broadly, this work highlights that the catalytic behavior of Cu cannot be understood solely in terms of static surface structures but instead reflects a dynamically evolving interface shaped by the interplay of hydrogen, oxidizing intermediates, and electrochemical history.

## Supplementary Material



## Data Availability

Computational datasets are available on Zenodo at 10.5281/zenodo.18514761

## Notes

Electrochemical mass spectrometry calibration procedures for H_2_ quantification under varying carrier gas compositions; determination of electrolyte layer thickness by EC-MS impulse measurements; cyclic voltammetry and potential pulse measurements on Cu(111) with and without O_2_ in the carrier gas; analysis of steady-state and transient H_2_ flux, charge deconvolution, and limits of detection; comparison of hydrogen evolution kinetics before and after O_2_ exposure; and grand canonical genetic algorithm sampling, surface phase free energies, adsorption configurations, and reaction energetics from DFT and ab initio molecular dynamics calculations (DOCX)
