# Supplementary material for: O2 Reduction Stimulates Adatom Generation on Cu(111) Catalyzing Hydrogen Evolution
Source: J Am Chem Soc. 2026 Feb 11;148(7):7401–14. doi: 10.1021/jacs.5c20244 (PMC12951440; doi:10.1021/jacs.5c20244)
Supplement: Supplementary file 1 [file ja5c20244_si_001.docx]

**Supporting Information for**

**O_2_ Reduction Stimulates Adatom Generation on Cu(111) Catalyzing Hydrogen Evolution**

David Raciti*^,1^, Zisheng Zhang*^2,3^, Ally Guo^1^and Thomas P. Moffat*^,1^

^1^Material Science and Engineering Division, National Institute of Standards and Technology, 100 Bureau Drive, Gaithersburg, MD 20899, USA

^2^SUNCAT Center for Interface Science and Catalysis, SLAC National Accelerator Laboratory, 2575 Sand Hill Road, Menlo Park, CA 94025, USA

^3^Department of Chemical Engineering, Stanford University, 443 Via Ortega, Stanford, CA 94305, USA

AUTHOR INFORMATION

ORCID:

David Raciti: 0000-0002-9580-4524

Zisheng Zhang: 0000-0002-4370-4038

Ally Guo: 0009-0000-0314-389X

Thomas P. Moffat: 0000-0003-4377-1692

*Corresponding Authors: David Raciti ([david.raciti@nist.gov](mailto:david.raciti@nist.gov)), Zisheng Zhang ([zishengz@stanford.edu](mailto:zishengz@stanford.edu)), Thomas P. Moffat ([thomas.moffat@nist.gov](mailto:thomas.moffat@nist.gov))

*Calibration of the electrochemical mass spectrometer for H_2_ quantification in varying carrier gas mixtures.*

Calibration of the electrochemical mass spectrometry (EC-MS) for quantitative determination of H_2_ via the 2 amu signal was tracked using both *ex-situ* and *in-situ* methods. Both have been discussed previously.^1,2^ However, the details of calibration are worth revisiting due to the introduction of O_2_ into the EC-MS carrier gas for select experiments as discussed below. To enable a general understanding of the relationship between carrier gas make-up and the H_2_ calibration curve, chronoamperometry was performed using a polycrystalline Pt crystal in 0.1 mol/L HClO_4 ­_with He as the carrier (**Figure S1**). The assumption was made that under pseudo steady-state conditions the charge consumed at the working electrode is due to the hydrogen evolution reaction (HER), with an offset in the x-intercept due to trace oxygen reduction as discussed previously.^2^ Therefore, the electrochemical current can be converted to a rate of H_2_ corresponding to the respective 2 amu signal current from the MS (**Figures S1a-S1b**). Linear regression can then be used to generate a calibration curve (**Figure S1c**). The slope, *k_ECMS_*, is used to convert the 2 amu signal (A) or integrated 2 amu signal (C) to a rate of H_2_ (mol/s) or a quantity of H_2_ (mol), respectively. Within this experimental platform the carrier gas was modulated to contain various partial pressures or ratios of He, N_2_, Ar, and O_2_ (**Figure S2**). Mild fluctuation in the slope of the calibration curve was observed over the course of an experiment and between days (e.g., from 3.65 mol cm^-2^ C^-1^ to 4.13 mol cm^-2^ C^-1^) even when the carrier gas wasn’t changed (**Figure S2a**). Note: the reported slopes in **Figure S2** are denoted as 1/*k_ECMS_*. In the case of He/Ar mixtures, *k_ECMS_* spanned from 3.50 mol cm^-2^ C^-1^ in He dominant mixes to 4.07 mol cm^-2^ C^-1^ in Ar dominant mixtures (**Figure S2b**). A small difference was observed between N_2_ and Ar as well (3.09 mol cm^-2^ C^-1^ vs. 3.43 mol cm^-2^ C^-1^, respectively, **Figure S2c**). However, the changes in *k_ECMS_* observed for these inert gas mixtures generally fall within the scattered observed in pure He.

Finally, and most importantly for the discussion herein, O_2_ was found to perturb the MS sensitivity the most compared to the other carrier gas mixtures studied. The presence of O_2_ results in the ORR, which complicates the assumption that 100 % of the charge is being consumed by the HER. Fortunately, for the applied potentials used in determination of *k_ECMS_*, the ORR is mass transport limited. Therefore, the current/charge consumed for ORR should not change substantially under such conditions (assuming the number of electrons delivered per O_2_ remains constant). The presence of ORR manifests as a shift in the x-intercept of the linear fit used to determine *k_ECMS_* (as indicated in **Figures S1-S3**). As mentioned above, even without O_2_ in the carrier the intercept of the fit does not pass through zero which is likely a result of parasitic ORR from trace O_2_ which can infiltrate the electrochemical cell through connections and gaps. When the carrier gas contained 17 % O_2_ the sensitivity for H_2_ decreased by 30 % from 4.125 mol cm^-2^ C^-1^ to 6.135 mol cm^-2^ C^-1^ (**Figure S2d**). When O_2_ was eliminated from the carrier, a recovery in the H_2_ sensitivity to 3.539 mol cm^-2^ C^-1^ was observed. A similar, but larger fall-off was observed for 30 % O_2_ in the carrier where a 50 % loss in sensitivity was observed.

As demonstrated above, the mass spectrometer (quadrupole with Faraday cup and electron multiplier) sensitivity has multiple influences that make it prone to random drift, including time of operation/saturation, age of the filament, state of the electron multiplier or carrier gas mixture. Given the differential pumping and system pressure a higher signal to noise ratio was obtained with the electron multiplier off. Given the dynamic sensitivity of the MS, it was determined that use of a *k_ECMS_* generated using Pt could lead to errors in quantification for experiments on Cu(111). As such, while the Pt measurements enabled insight into the influence of carrier gas make-up on MS sensitivity, the same methodology was necessary to apply with the Cu(111) electrode to determine *k_ECMS_* during the experiment. **Figure S3** demonstrates the *in-situ* determination of *k_ECMS_* during potential pulse measurements (discussed below) on Cu(111) in 0.1 mol/L HClO_4_ using He (both before and after O_2_ was introduced) and various amounts of O_2_ in He (mass flow rate). The *k_ECMS_* reported in **Figure S3** were used to quantify 2 amu H_­2_ measured by EC-MS as detailed throughout the manuscript.

**Supporting Figures**


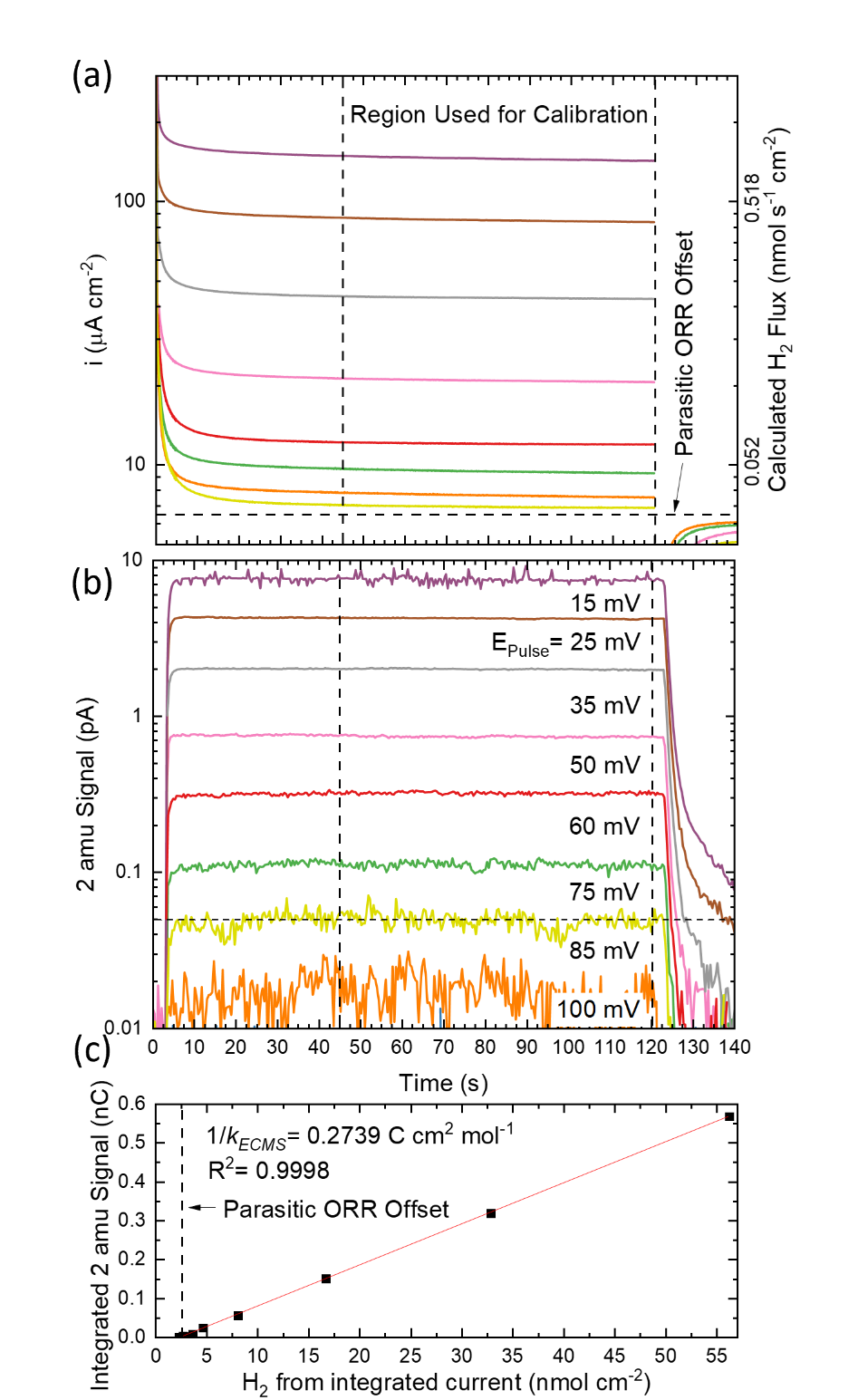


**Figure S1.** EC-MS potential pulse measurements using Pt in 0.1 mol L^-1^ HClO_4_ with He as the carrier. (a) The electrochemical current and corresponding (b) 2 amu signal were integrated in a steady state region for the series of applied potentials listed in (b) to generate a (c) linear calibration curve. A calibration curve offset was observed due to the parasitic ORR from the trace O_2_ present in the system. All potentials reported are positive and vs. the reversible hydrogen electrode (RHE). The rest potential between pulses was 0.2 V.


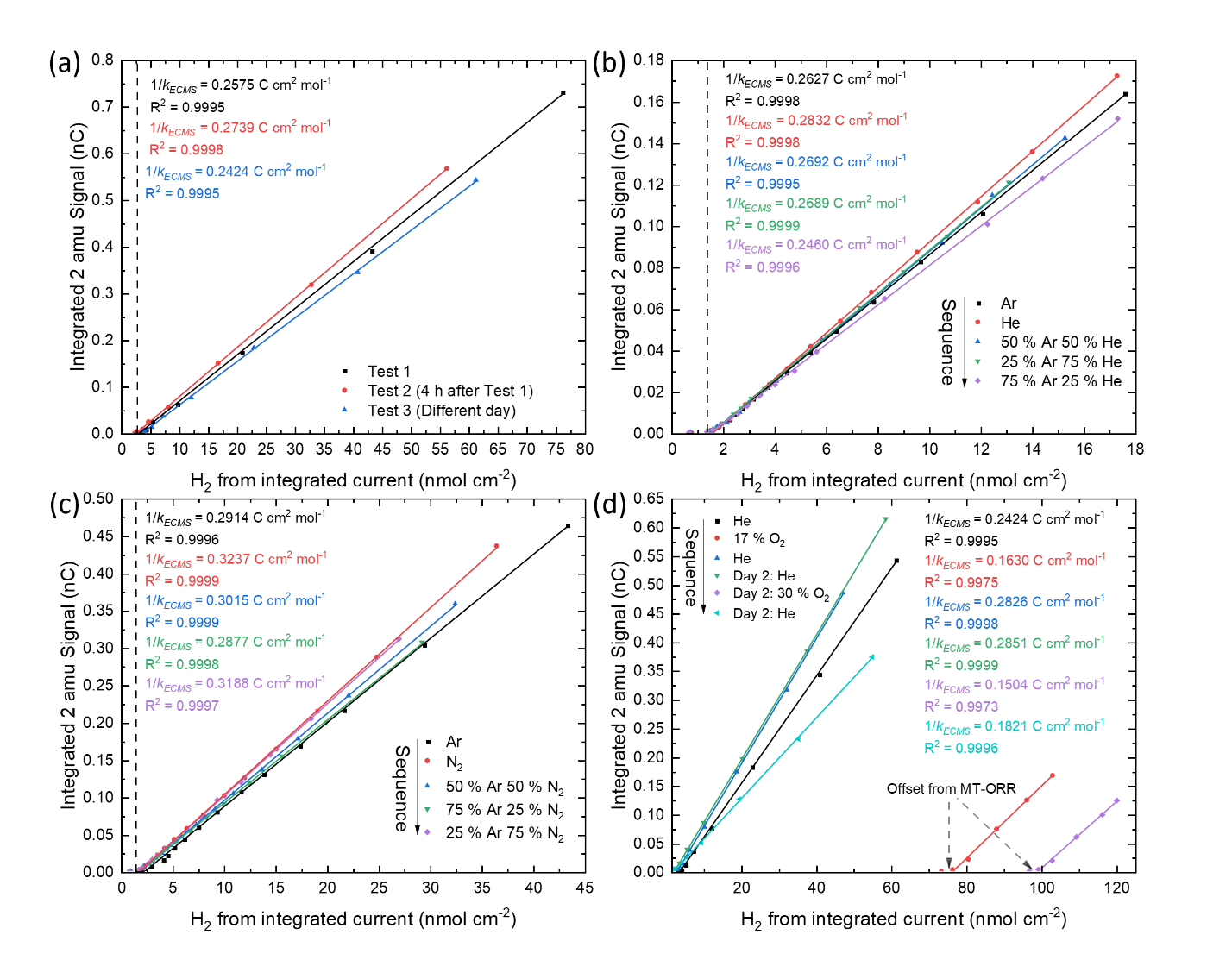


**Figure S2.** Calibration curves of the 2 amu signal (e.g., H_2_) using Pt (a) at different times with He as the carrier gas, (b) with mixtures of He and Ar as the carrier gas, (c) with mixtures of Ar and N_2_ as the carrier gas, and (d) with mixtures of O­_2_ and He as the carrier gas. The dashed lines indicate the offset due to parasitic ORR.


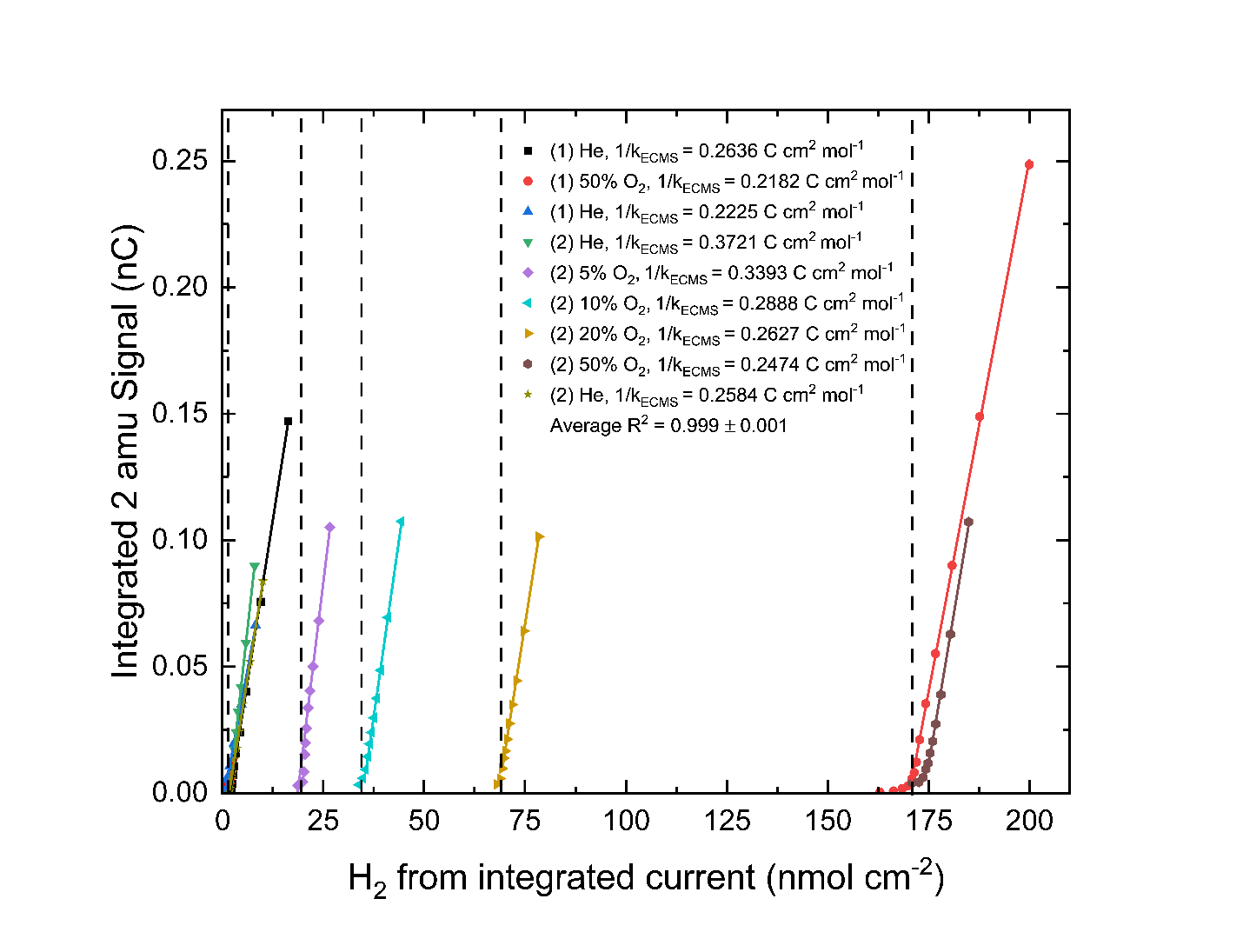


**Figure S3.** Calibration curves of the 2 amu signal (e.g., H_2_) using Cu(111) in 0.1 mol L^-1^ HClO_4_ with varying mixtures of He and O­_2_ as the carrier gas. The vertical dashed lines represent the approximate x-intercept which is due to mass transport limited current from the ORR (parasitic when 100 % He is used as the carrier).


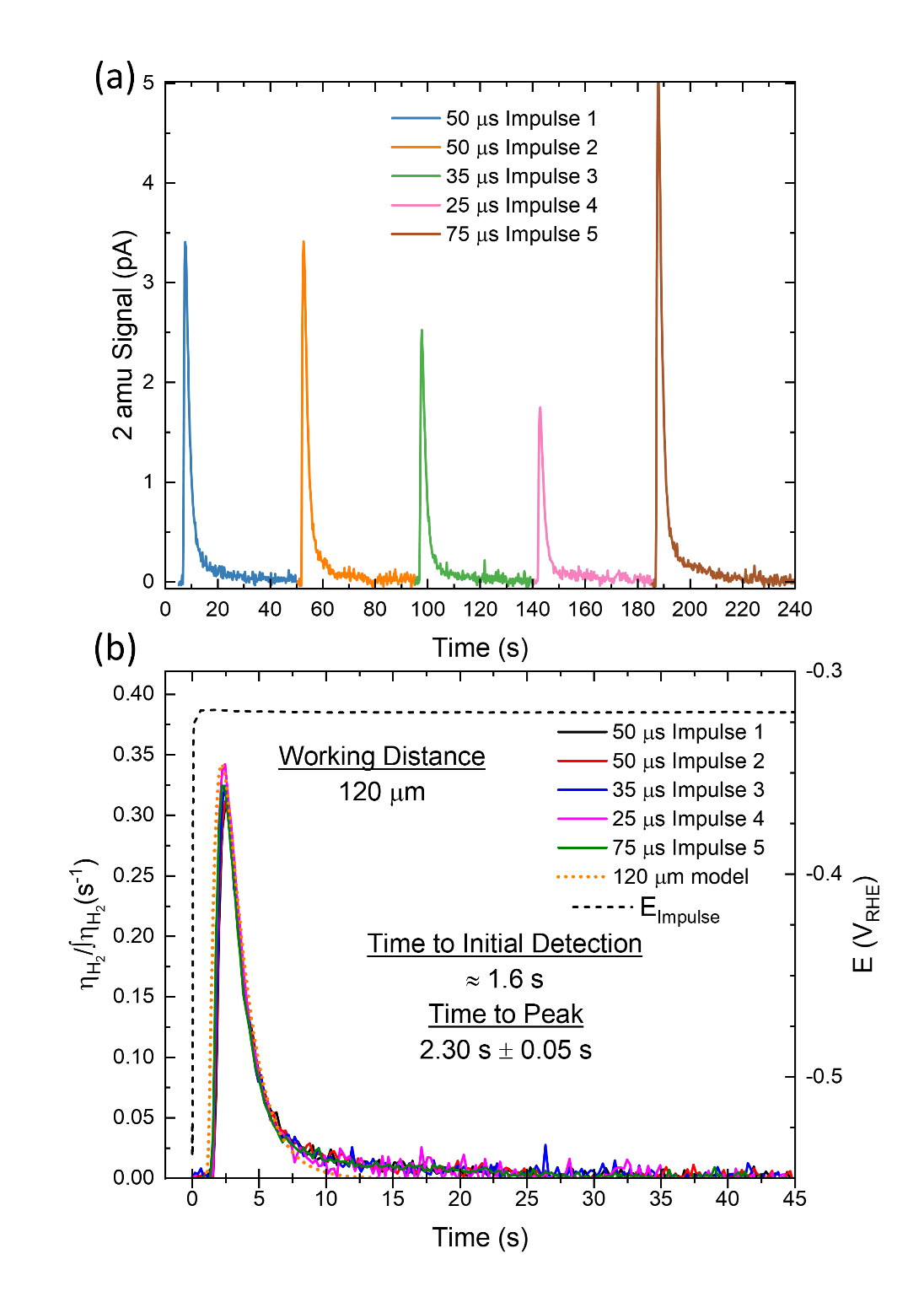


**Figure S4.** In-situ EC-MS impulse measurement to determine the thickness of the electrolyte layer (working distance). (a) Impulses were performed by pulsing the potential to -0.54 V_RHE_ for the duration (25 µs to 75 µs) indicated in the legend. In between impulses the Cu(111) disk was held at -2 µA for 45 s. The flat background produced by the -2 µA hold was background subtracted. (b) The integrated area during the pulse was used to normalized the 2 amu signal and the normalized signal was fit to a model developed by Krempl et al. determining the electrolyte thickness to be 120 μm.^3^


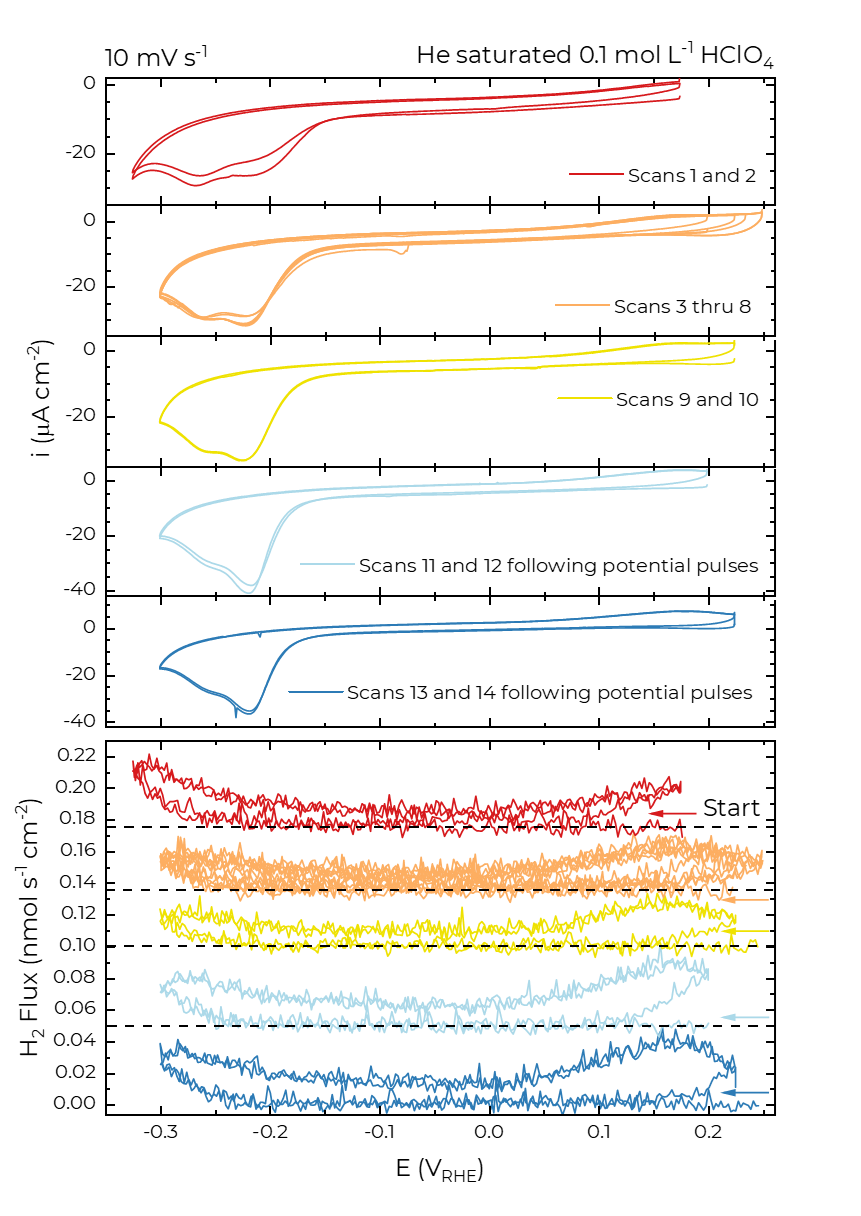


**Figure S5.** Tracking the development of the hydride formation peaks via cyclic voltammetry using the EC-MS. All scans were performed at 10 mV s^-1^ in He saturated 0.1 mol L^-1^ HClO_4_. The scan numbers notate the scan sequence from initiation of the experiment, although scans 11 onward were obtained after the pulse sequence presented in **Figures 5a-5b**.


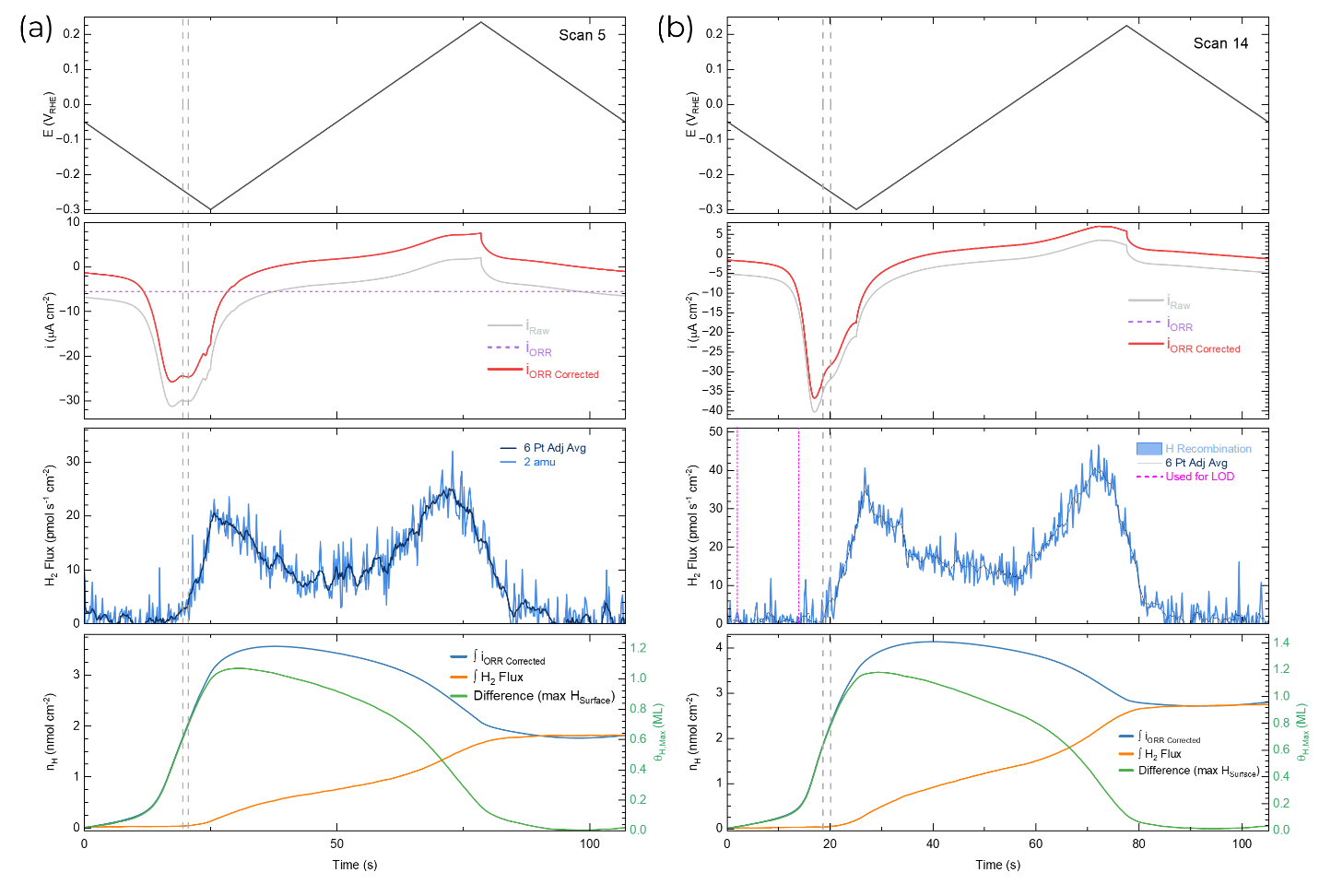


**Figure S6.** Traces of potential, current, and H_2_ flux vs. time from the (a) 5^th^ and (b) 14^th^ CVs scans plotted in **Figure 1** (also **Figure S5**). Integration of current and H_2_ flux as well as their difference in the form of atomic hydrogen (n_H_, nmol cm^-2^) demonstrates an estimated coverage of H_ads_ on the Cu surface as a function of potential (bottom panel). The magenta dashed vertical lines (b) serve as an example of the baseline signal used to determine the limit of detection (LOD) of the EC-MS. The LOD was used to determine the onset potential window of HER.


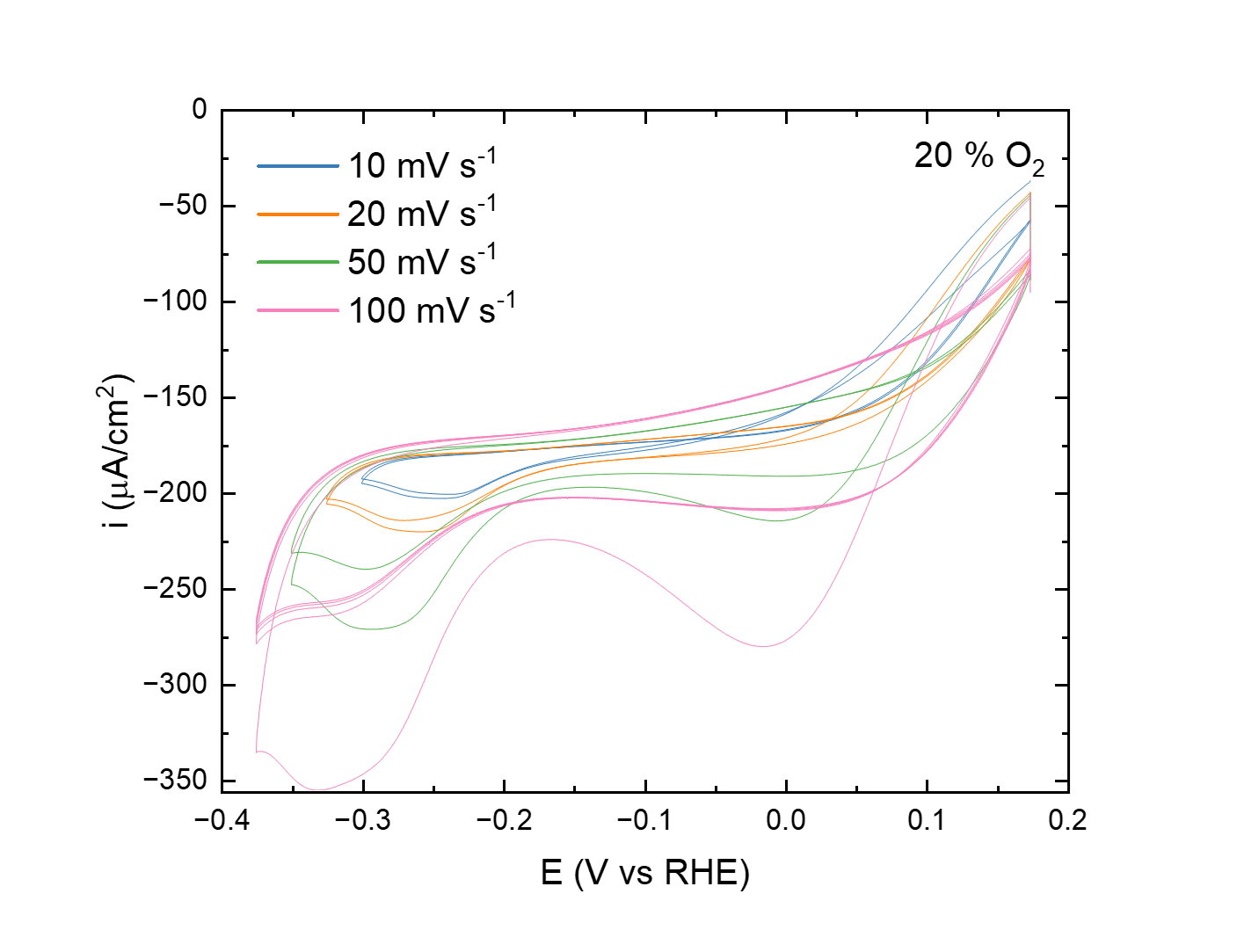


**Figure S7.** Voltammetry at different scan rates with 20 % O2 mixed with the He carrier gas.


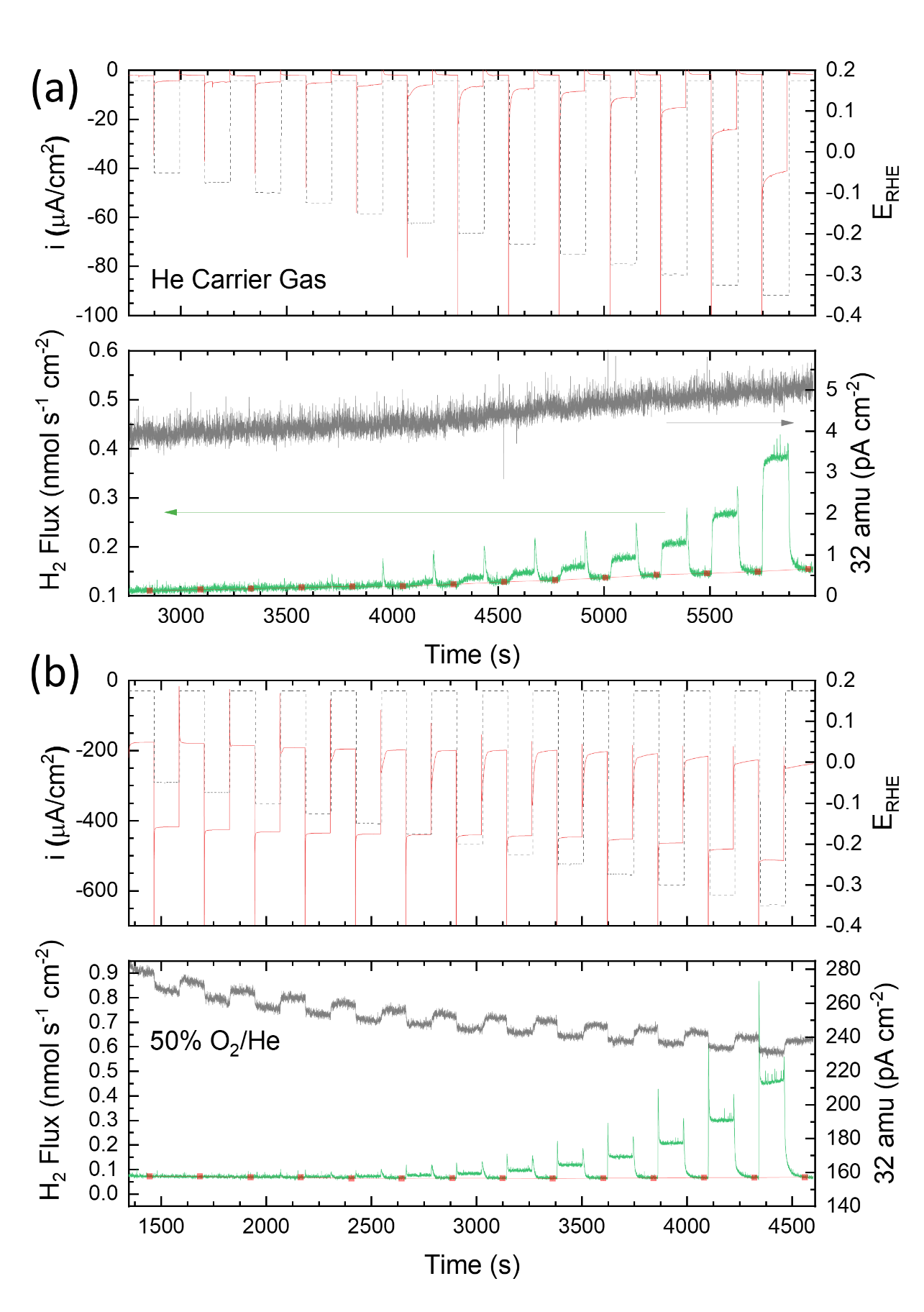


**Figure S8.** Potential pulse measurements on Cu(111) in 0.1 mol L^-1^ HClO_4_ with (a) He or (b) 50 % O_2_ in He as the carrier gas. The points in the baseline trace are the anchor points for the H_2_ flux baseline curve, which was determined by averaging the raw signal for 2 s.


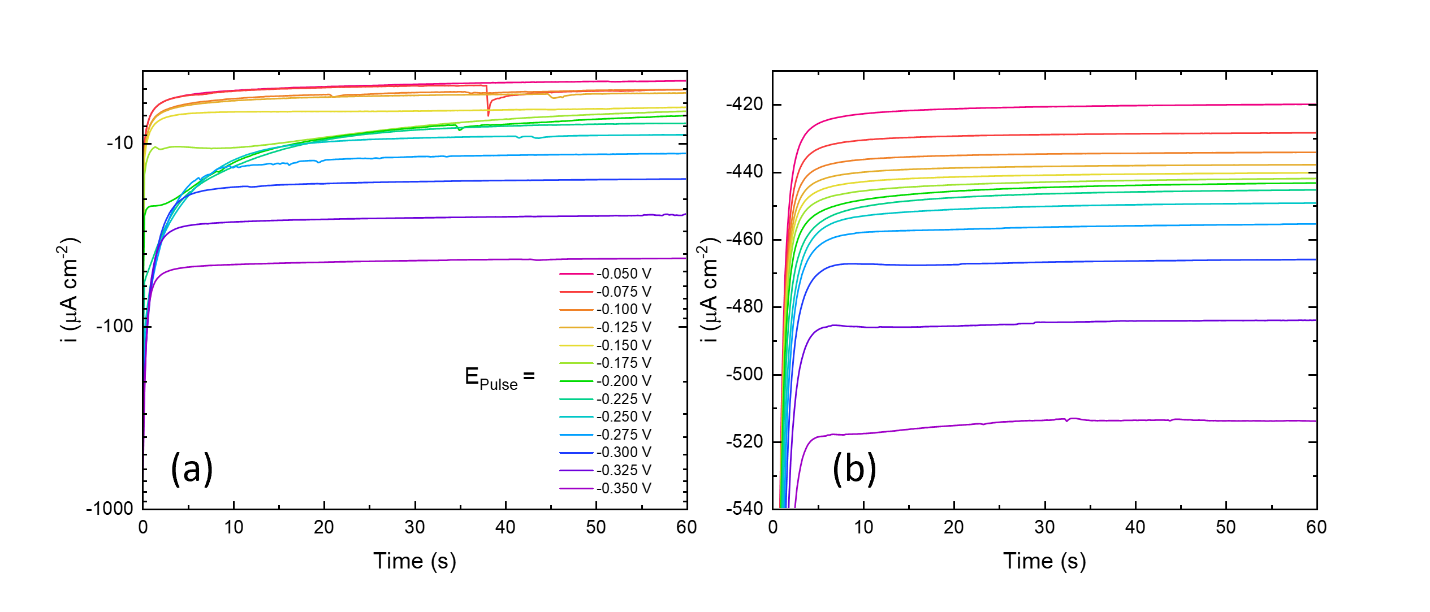


**Figure S9.** Current transients during E_pulse_ measurements on Cu(111) in 0.1 mol L^-1^ HClO_4_ with (a) He or (b) 50 % O_2_ in He as the carrier gas. This data is a zoomed in (and log scale for a) representation of **Figure 5a and 5c**.


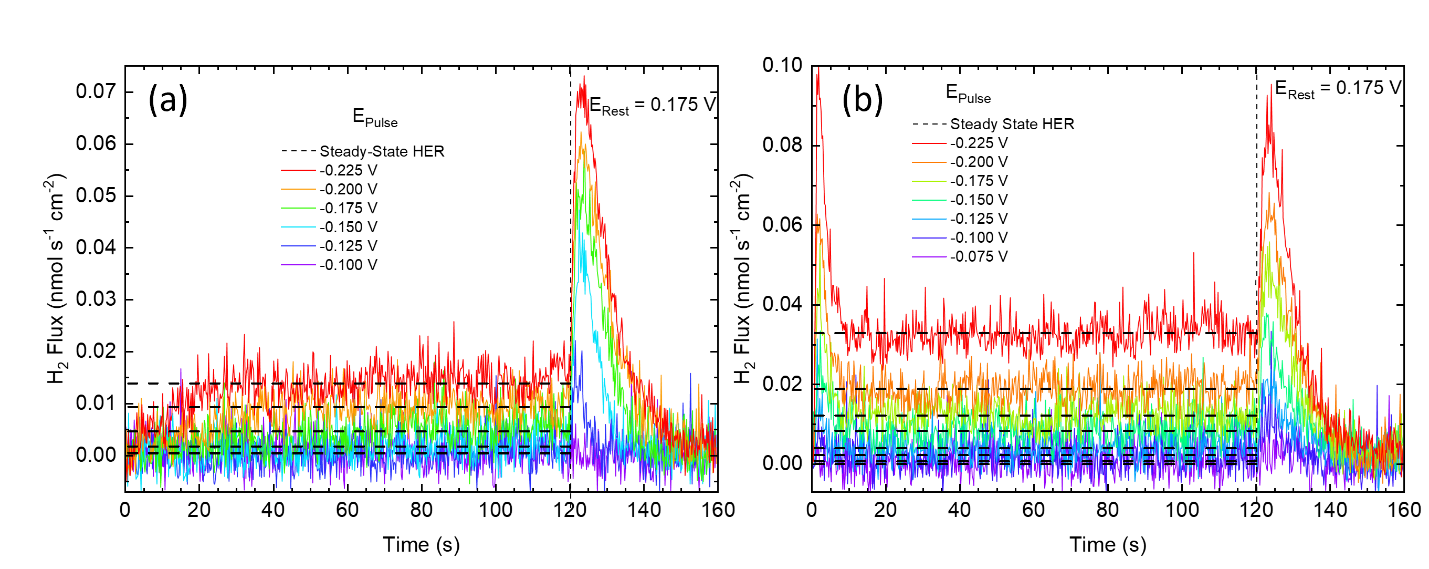


**Figure S10.** H_2_ flux during select E_Pulse_ on Cu(111) in 0.1 mol L^-1^ HClO_4_ with (a) He or (b) 50 % O_2_ in He as the carrier gas. This data is a subset of **Figure 5b and 5d**.


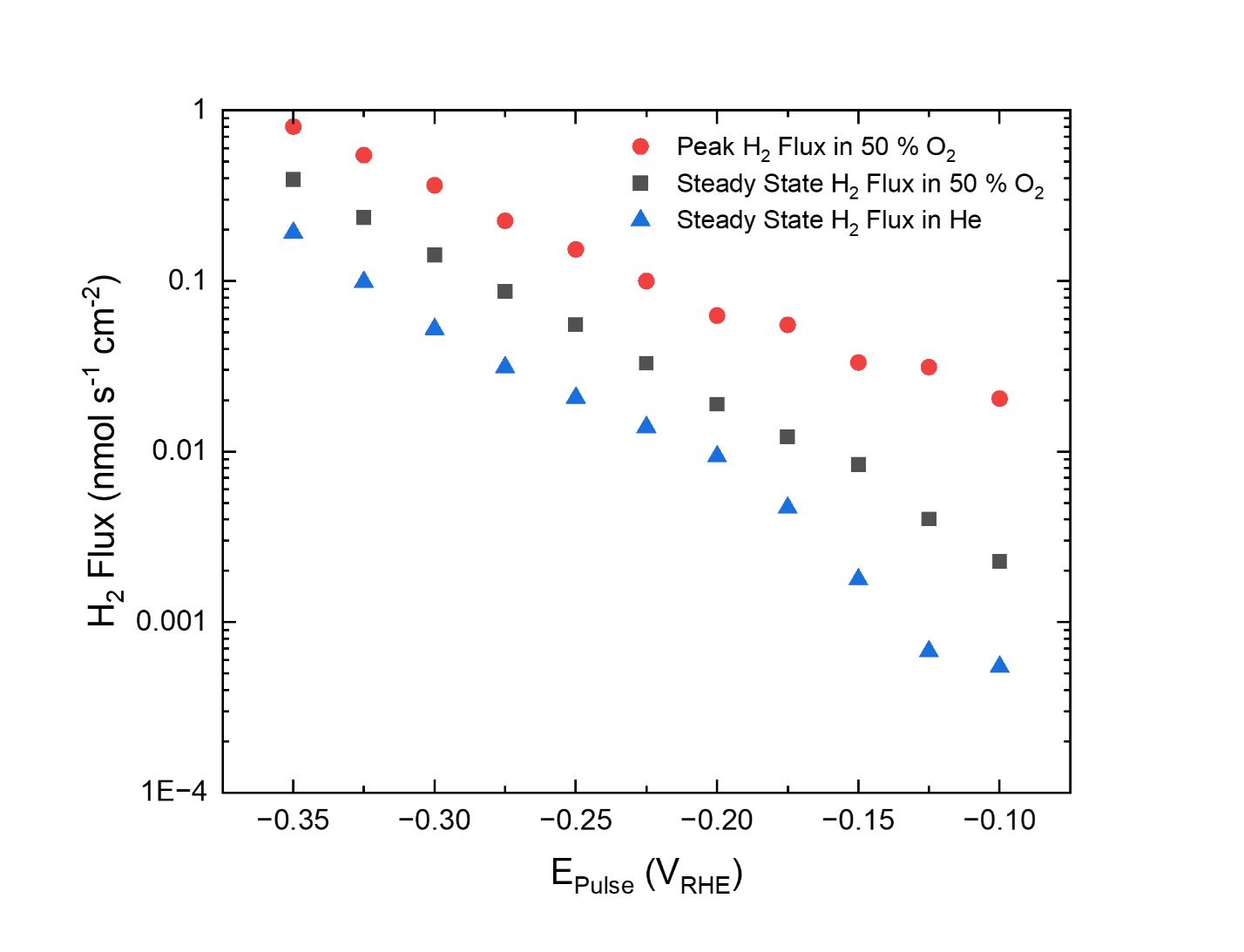


**Figure S11.** Comparison of peak H_2_ flux and the steady-state H_2_ flux during E_Pulse_ of the potential pulse measurements. The peak H_2_ flux refers to the transient spike observed in the initial 5 s of the measurement.


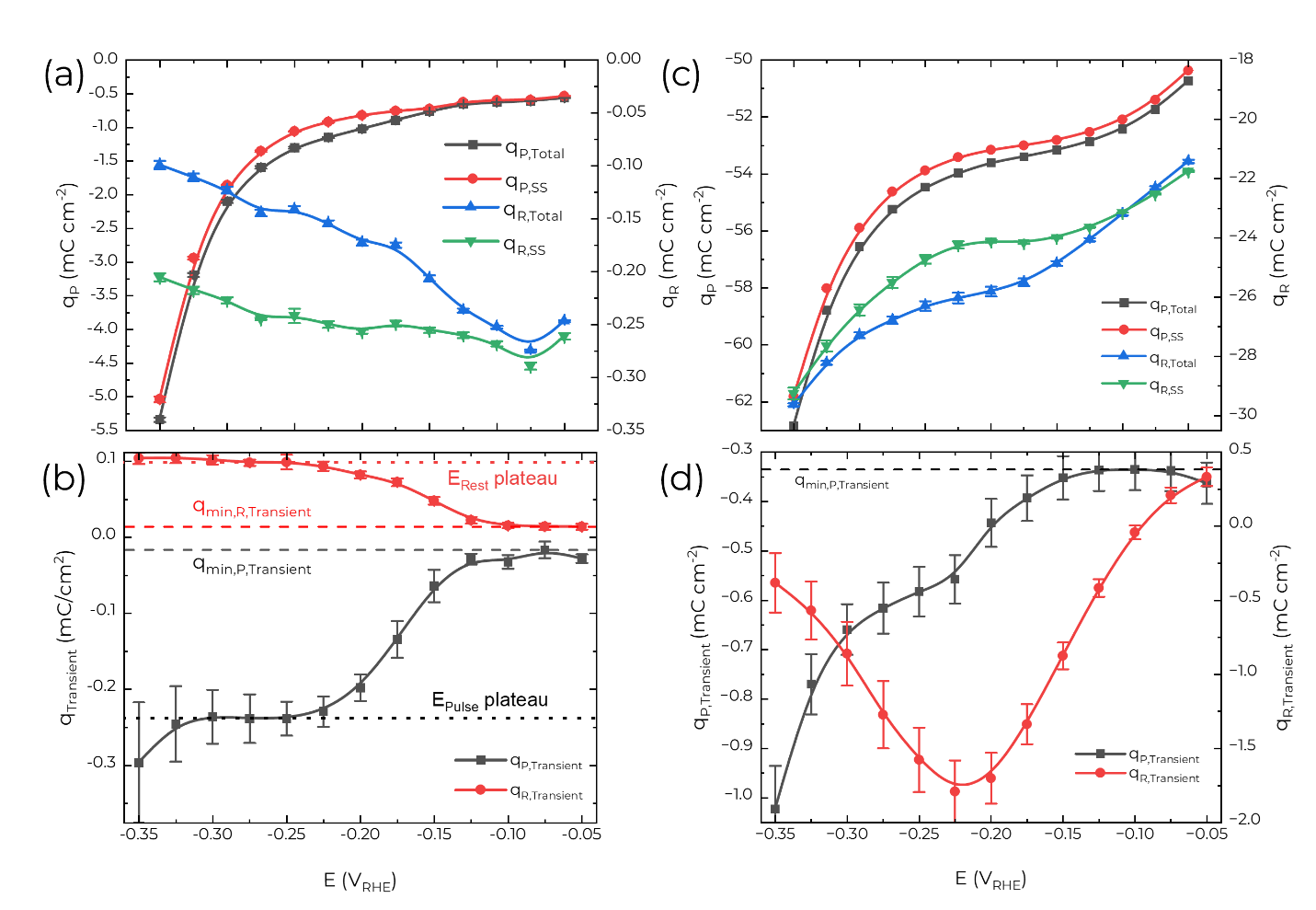


**Figure S12.** Total charge and deconvolution of (a,c) steady state charge and (b,d) transient charge (where q_Total_ = q_SS_ + q_Transient_) during potential pulse measurements in (a-b) He or (c-d) 50 % O_2_. The plateaus for E­_Rest_ and E_Pulse_ were found by averaging the charges for E_Pulse_ of -0.25 V, -0.275 V, and -0.3 V.


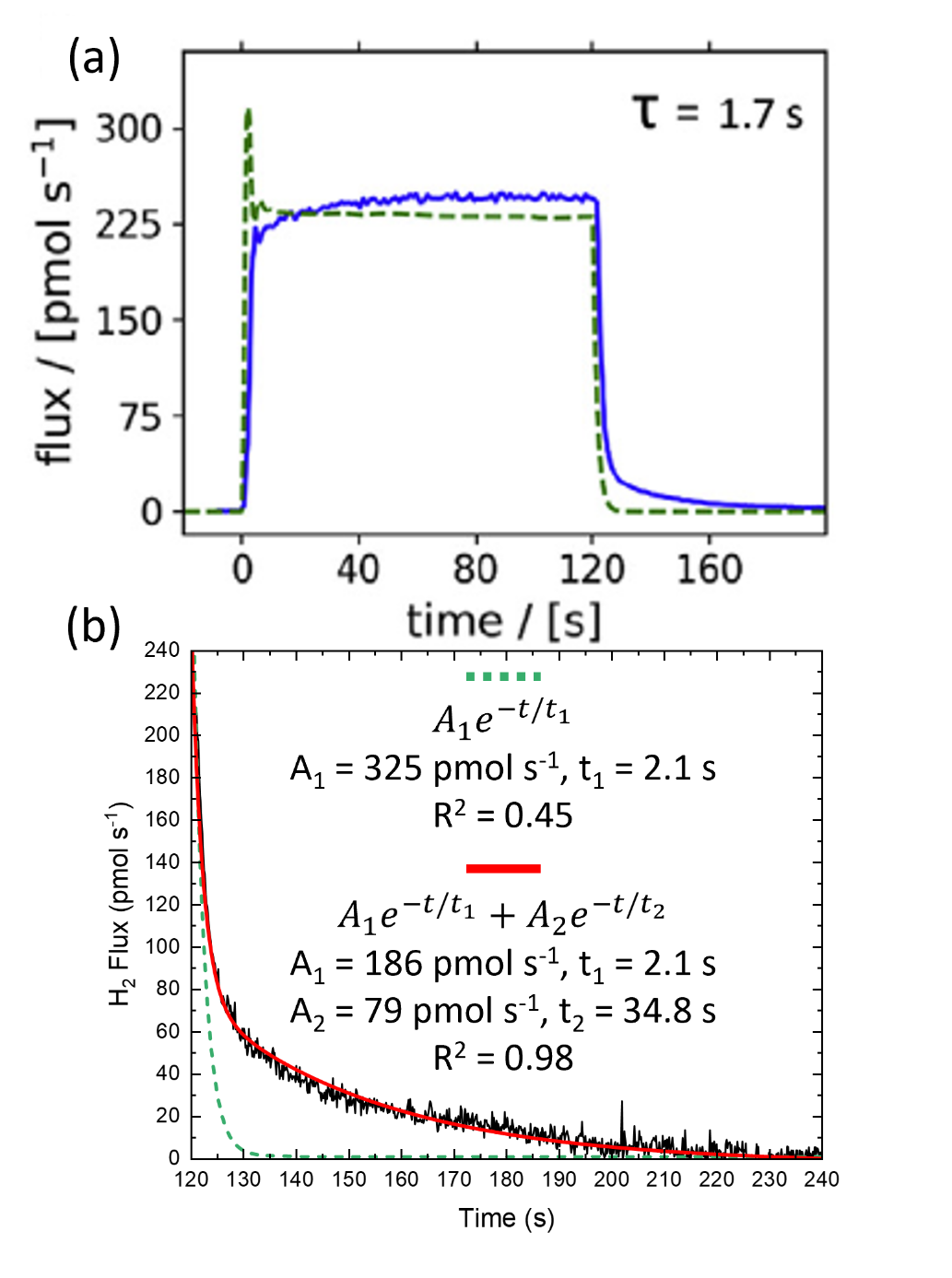


**Figure S13.** (a) Hydrogen flux measured by the EC-MS (blue line) and simulated flux assuming 100 % faradaic efficiency for the hydrogen evolution reaction (green dashes) during constant potential electrolysis on Pt in 1 mol L^-1^ HClO_4_ at 0.035 V vs. RHE from Trimarco et al.^4^ Reproduced from Ref. [4]: Electrochimica Acta 2018, 283, 1732–1740. Copyright © 2018 Elsevier Ltd. Reproduced with permission. (b) Fitting the empirically observed decay of H_2_ flux fro (a) with one exponential decay term (green dash) or two exponential decay terms of a control Ag experiment detailed in previous work.^2^


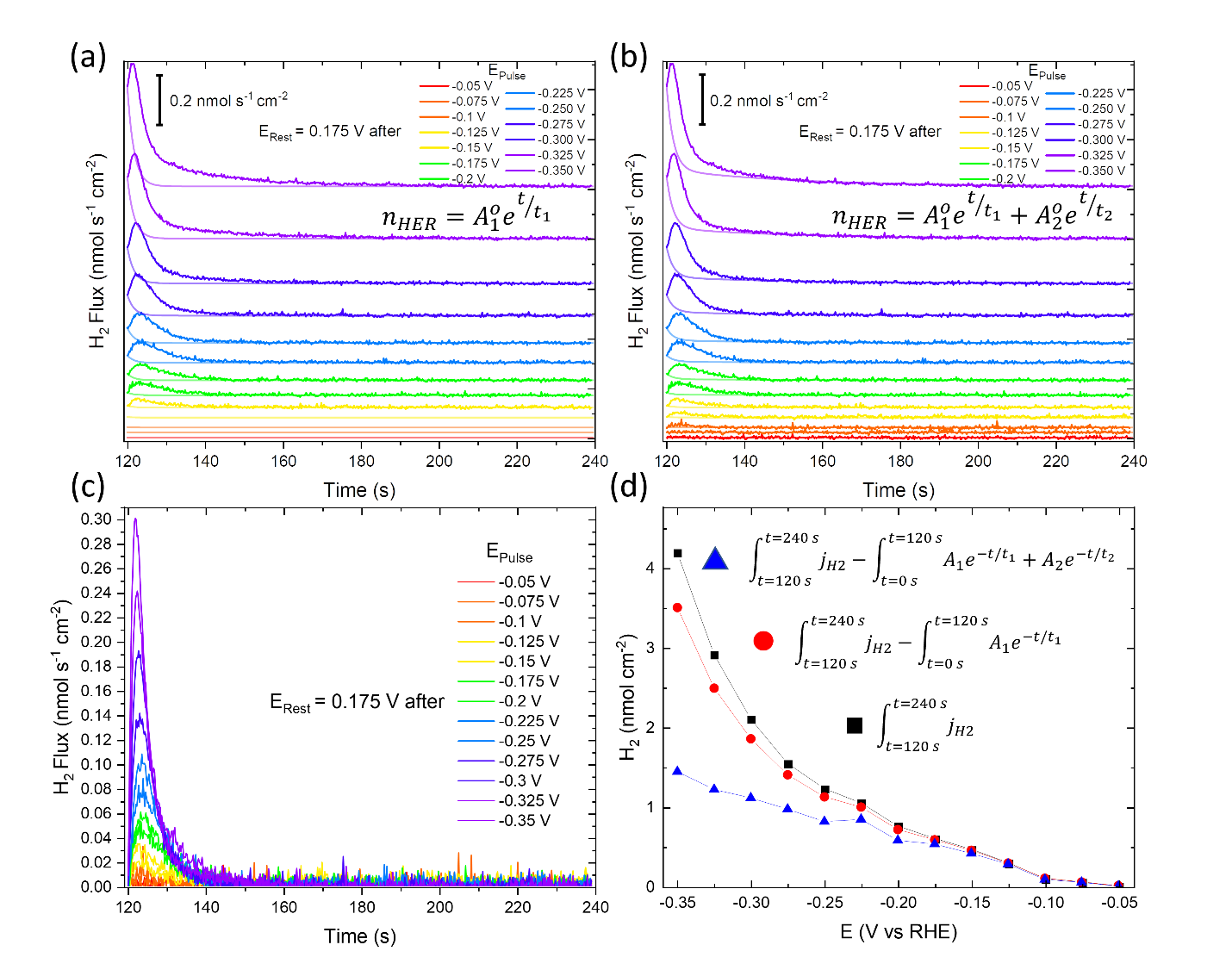


**Figure S14.** Fits for H_2_ flux measured by the EC-MS during the E_Rest_ of potential pulse measurements on Cu(111) with 50 % O_2_ saturated 0.1 mol L^-1^ HClO_4_ using (a) one exponential decay term or (b) two exponential decay terms. (c) Resulting H_2_ flux following subtraction of the fit shown in (b). (d) Integrated quantities of H_2_ from the unfit or fit H_2_ flux (j_H2_) data during E_Pulse_.


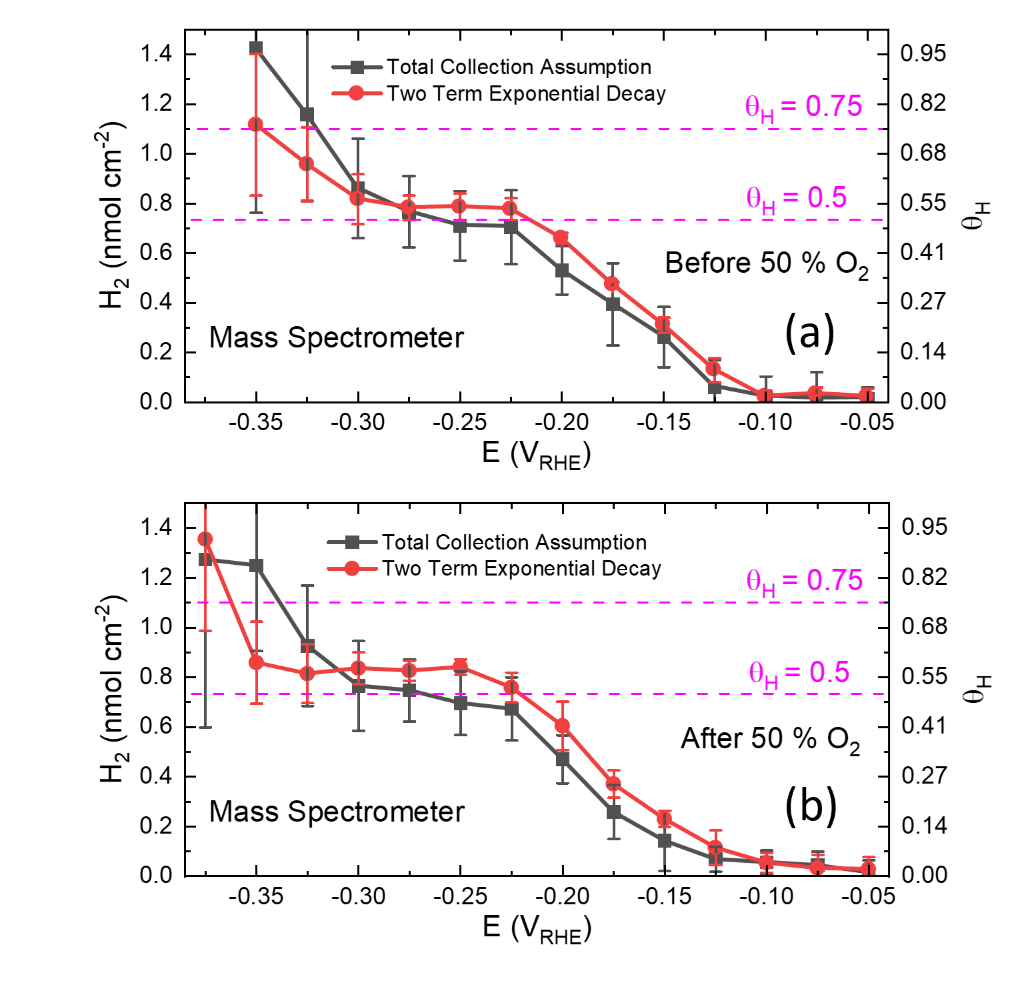


**Figure S15.** Comparison between the total collection assumption method and the two term HER exponential decay method to determine the quantity of H_2_ that can be ascribed to hydride decomposition (a) before and (b) after exposure to 50 % O_2_ in He.


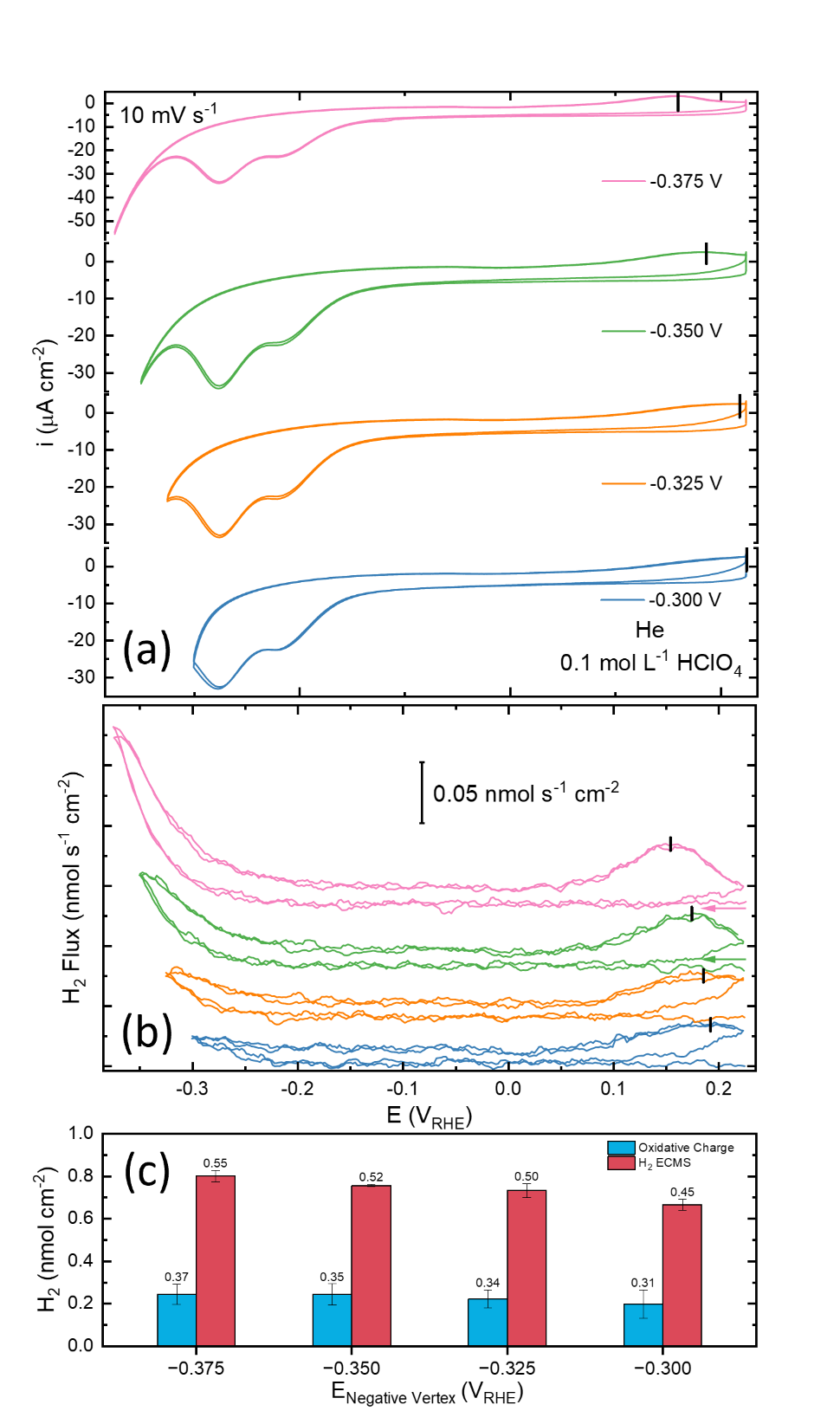


**Figure S16.** (a) Cyclic voltammetry at 10 mV s^-1^ in 0.1 mol L^-1^ HClO_4_ and corresponding (b) H_2_ flux for progressively more negative vertex potentials (-0.3 V, -0.325 V, -0.35 V, and -0.375 V) after exposure to 50 % O_2_. Two scans were collected for each negative vertex potential. The vertical black lines in (a) and (b) indicate the approximate peak potential for the oxidative peak and hydride decomposition, respectively. The H_2_ flux and oxidative peak (including the continued oxidation at the beginning of the second negative going sweep) were (c) integrated and converted to H (nmol cm^-2^), the labels above the bar graph represent the θ_H_.


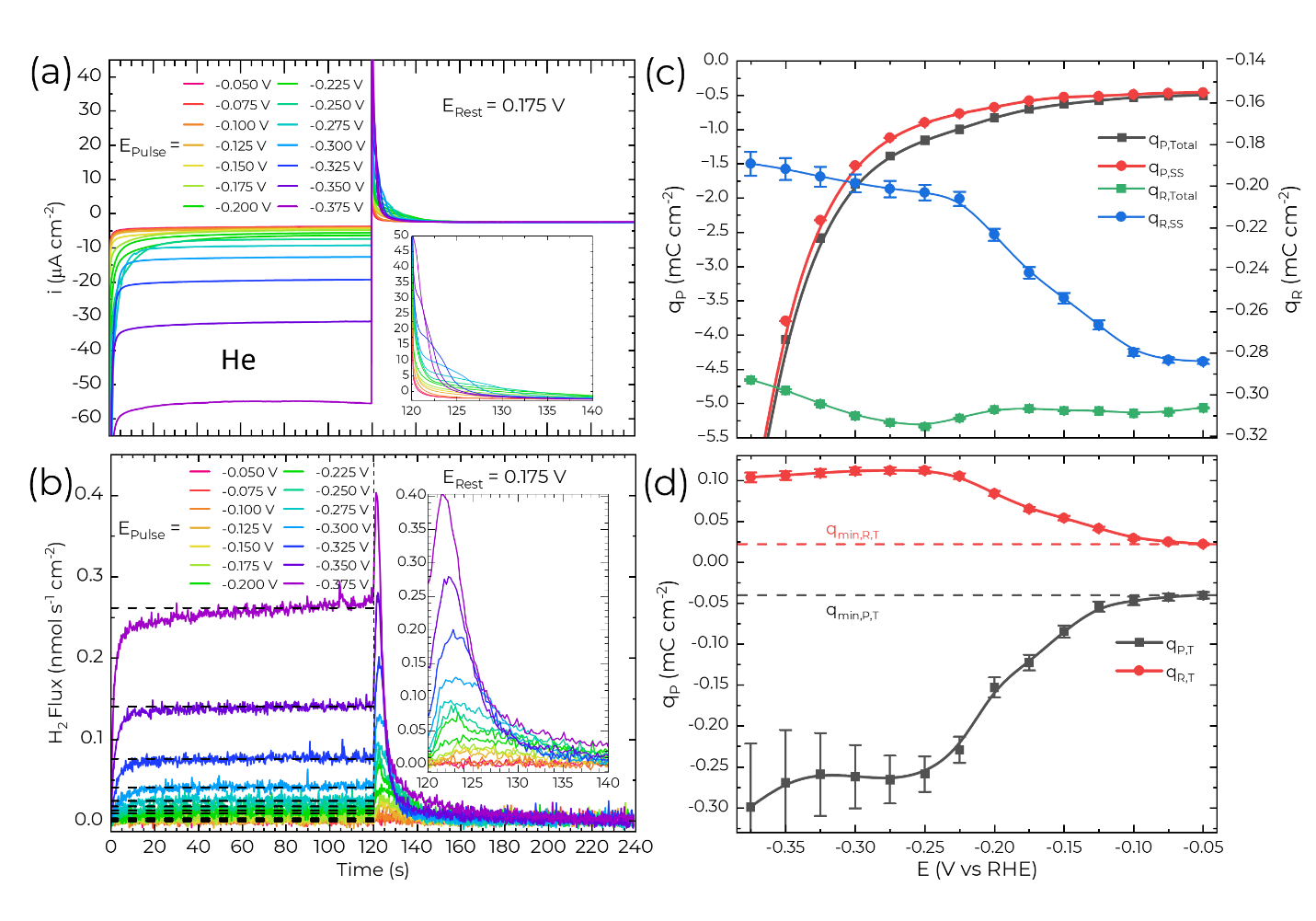


**Figure S17.** (a) Current (b) H_2_ flux, and (c-d) charges measured during potential pulse measurements on Cu(111) in He saturated 0.1 mol L^-1^ HClO_4_ following measurements in 50 % O_2_ saturated 0.1 mol L^-1^ HClO_4._


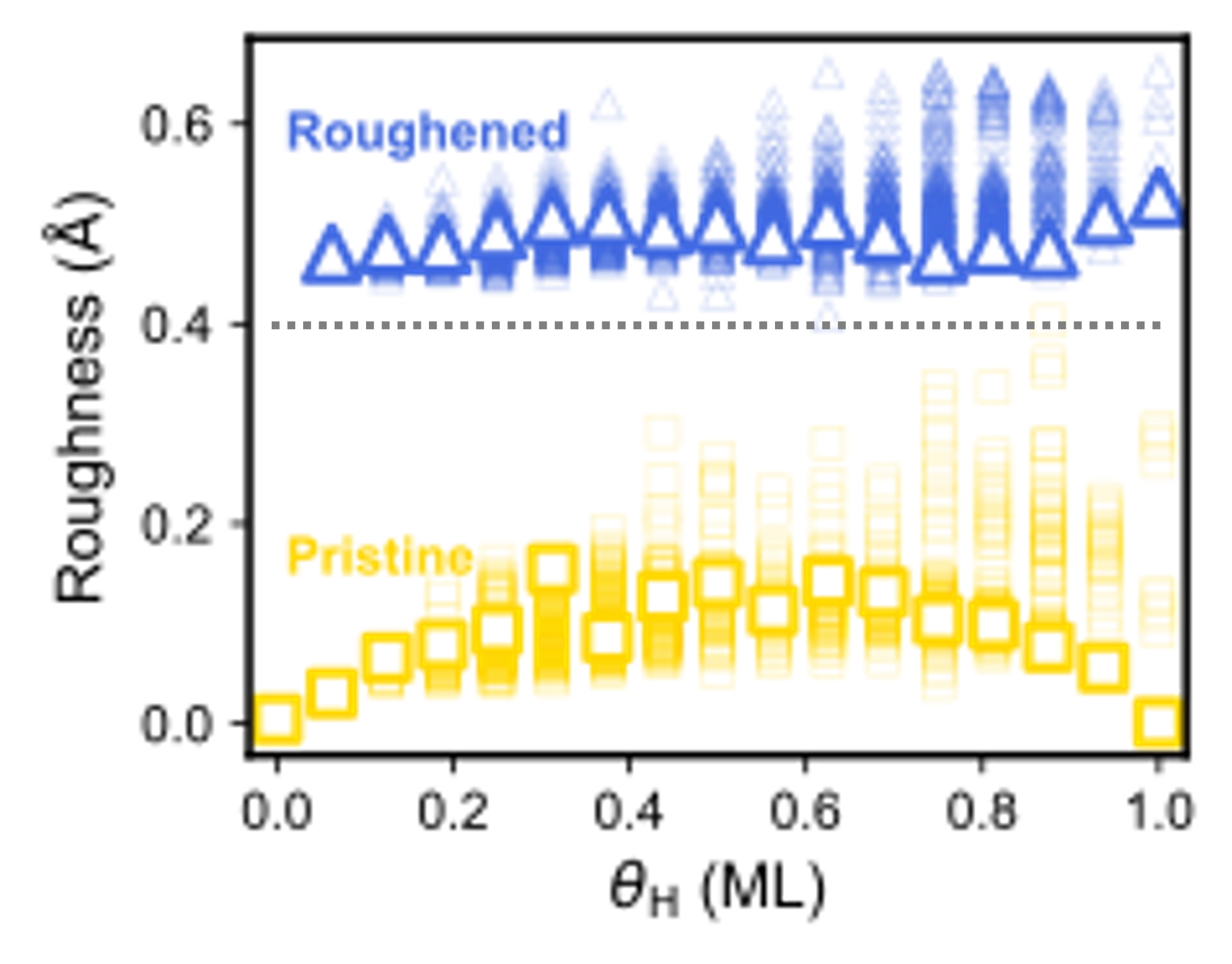


**Figure S18.** Distribution of samples from the (un)constrained grand canonical genetic algorithm minima searches. The grey dotted line marks the surface roughness threshold used in the constrained searches.


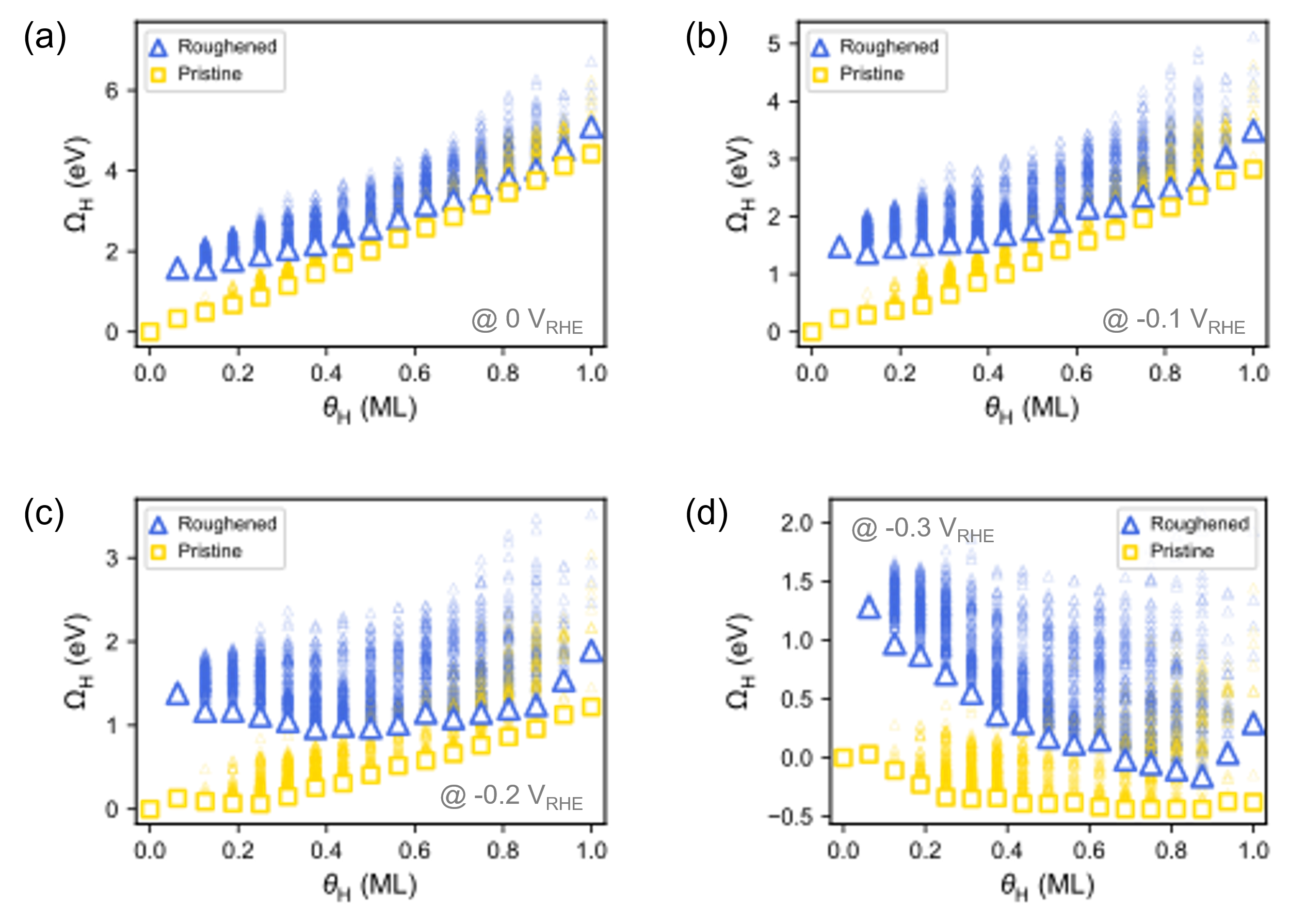


**Figure S19.** Grand canonical free energy of all surface phases in the final grand canonical ensemble, at potentials of (a) 0 V, (b) -0.1 V, (c) -0.2 V, and (d) -0.3 V, on RHE scale.


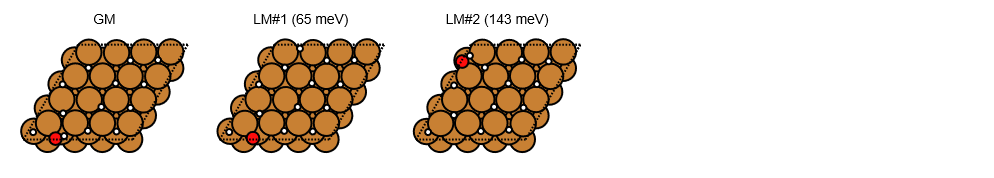


**Figure S20.** Global minima and low-energy local minima configurations of OH adsorption on the pristine hydride surface. Energies relative to the most stable configuration are labeled in the parenthesis.


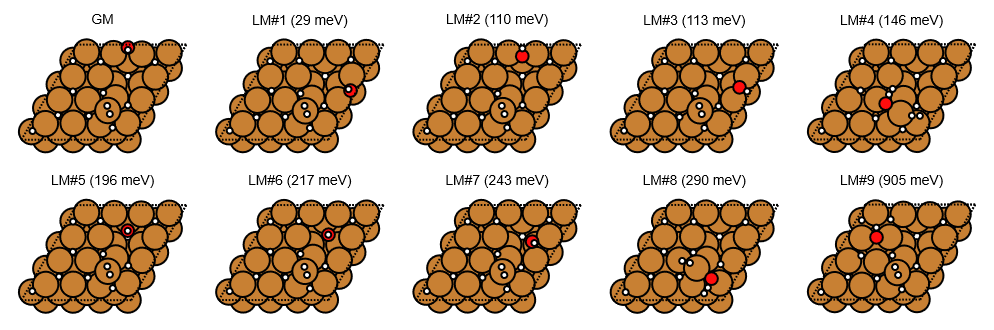


**Figure S21.** Global minima and low-energy local minima configurations of OH adsorption on the roughened hydride surface. Energies relative to the most stable configuration are labeled in the parenthesis.


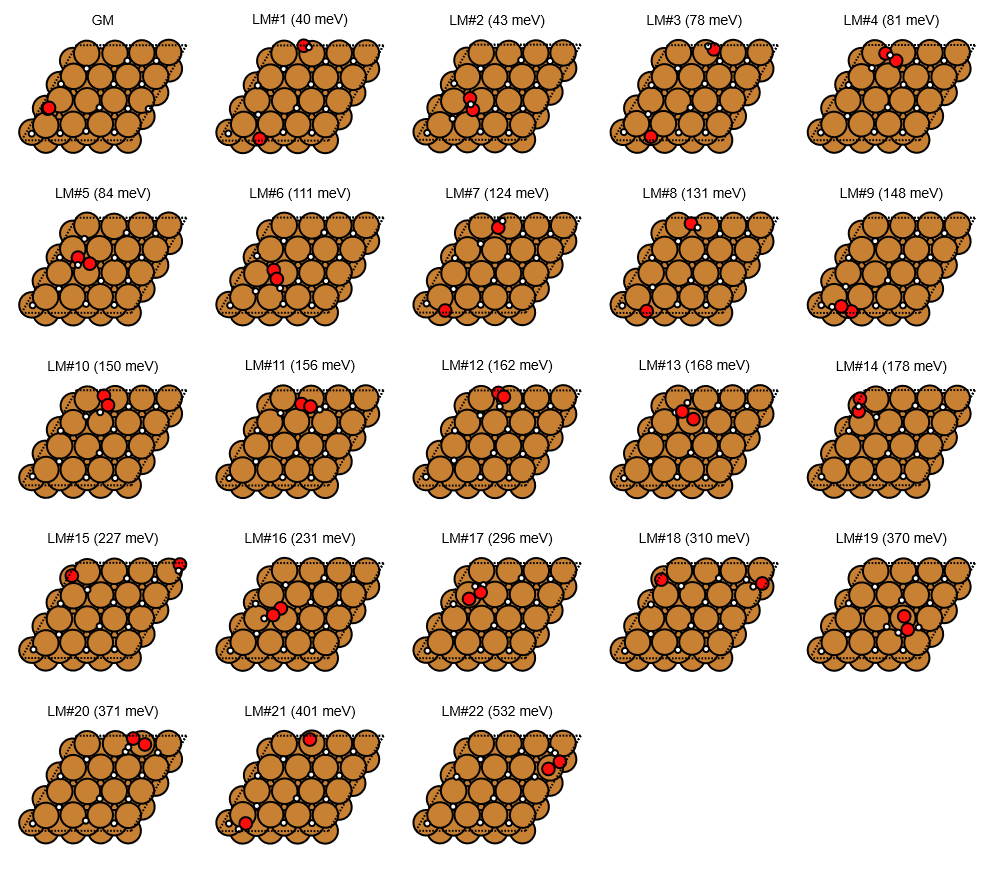


**Figure S22.** Global minima and low-energy local minima configurations of OOH adsorption on the pristine hydride surface. Energies relative to the most stable configuration are labeled in the parenthesis.


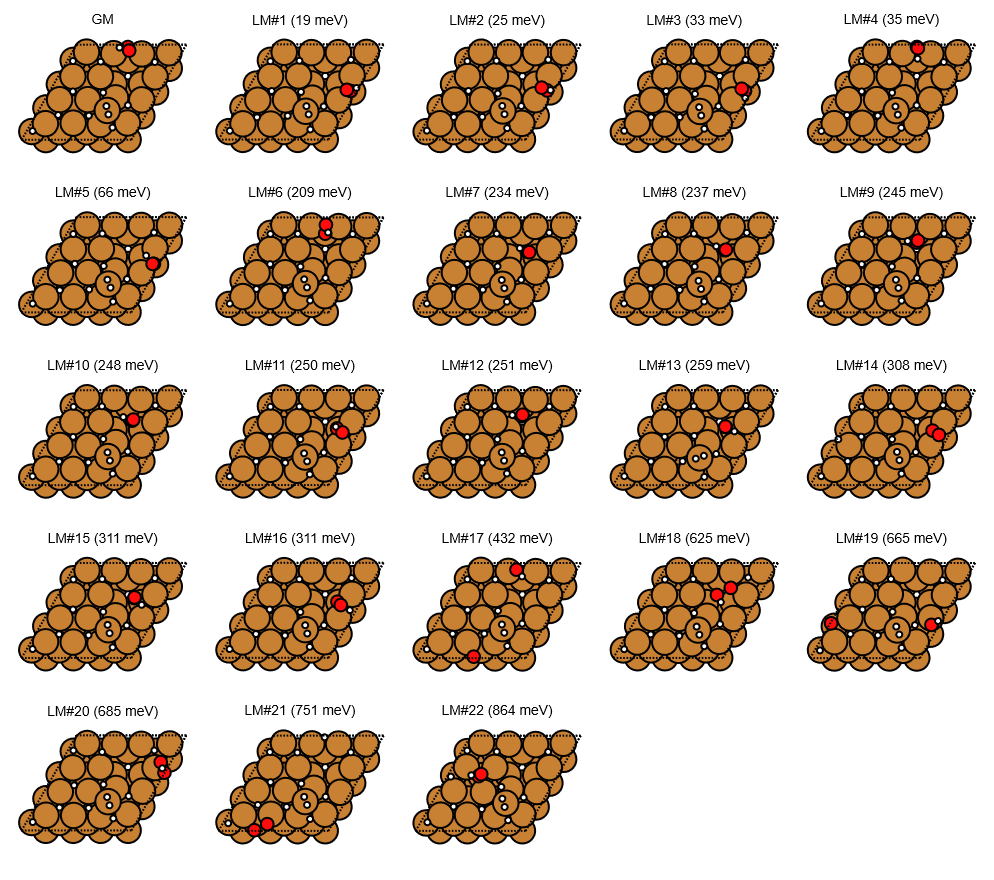


**Figure S23.** Global minima and low-energy local minima configurations of OOH adsorption on the roughened hydride surface. Energies relative to the most stable configuration are labeled in the parenthesis.


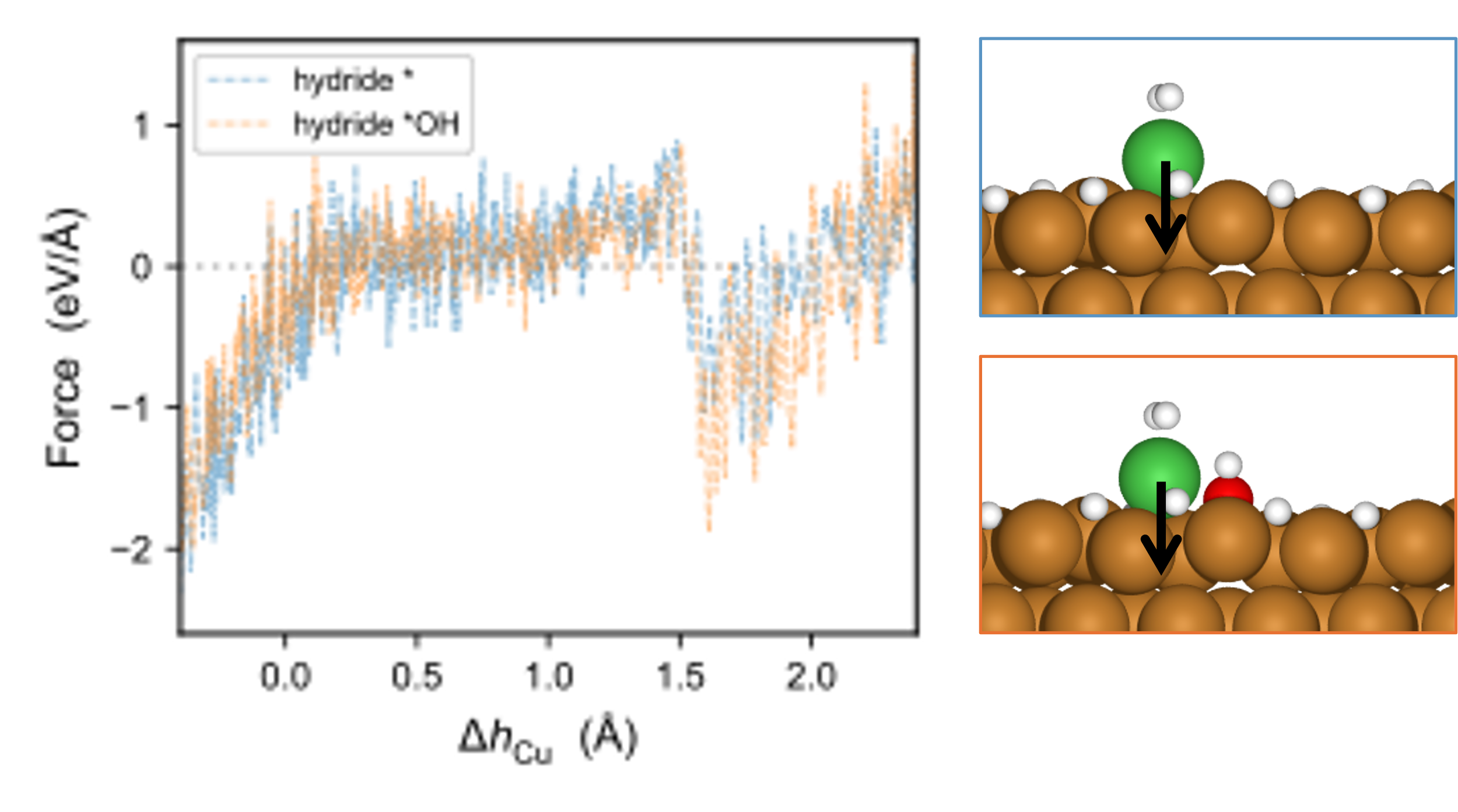


**Figure S24.** Evolution of the forces along the reaction coordinate during the constrained ab initio molecular dynamics simulations. The reaction coordinate for surface pristine-to-roughened transition is chosen as the height of the Cu adatom, as is shown on the right. Atomic color code: H – white, O – red, pristine Cu – brown, roughened Cu adatom – green.


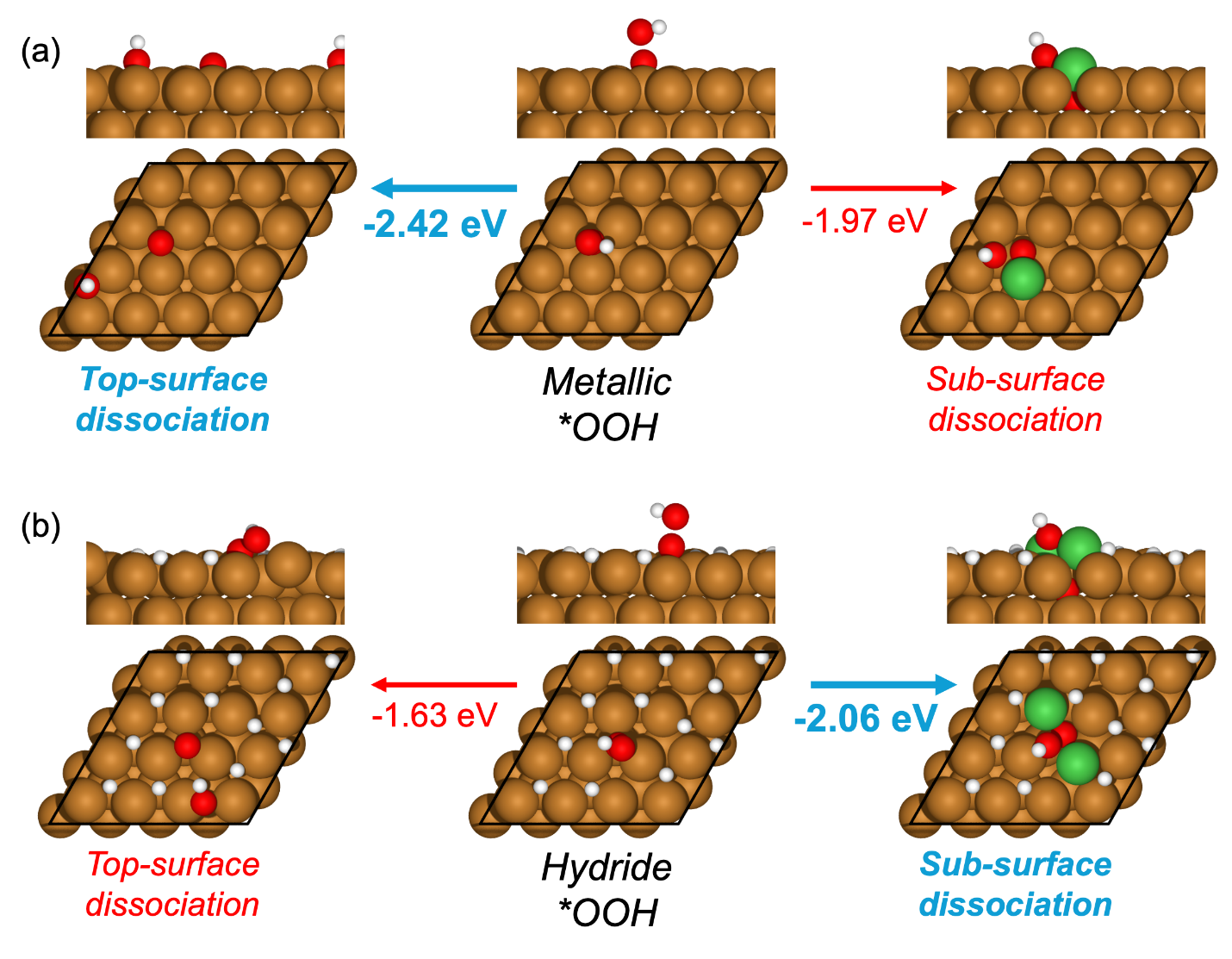


**Figure S25.** Reaction energetics of top-surface and sub-surface*OOH dissociation pathways on (a) metallic or (b) hydride surfaces. The favorable and the unfavorable pathways are in blue and red, respectively. Color code: H – white, O – red, pristine Cu – brown, roughened Cu – green.


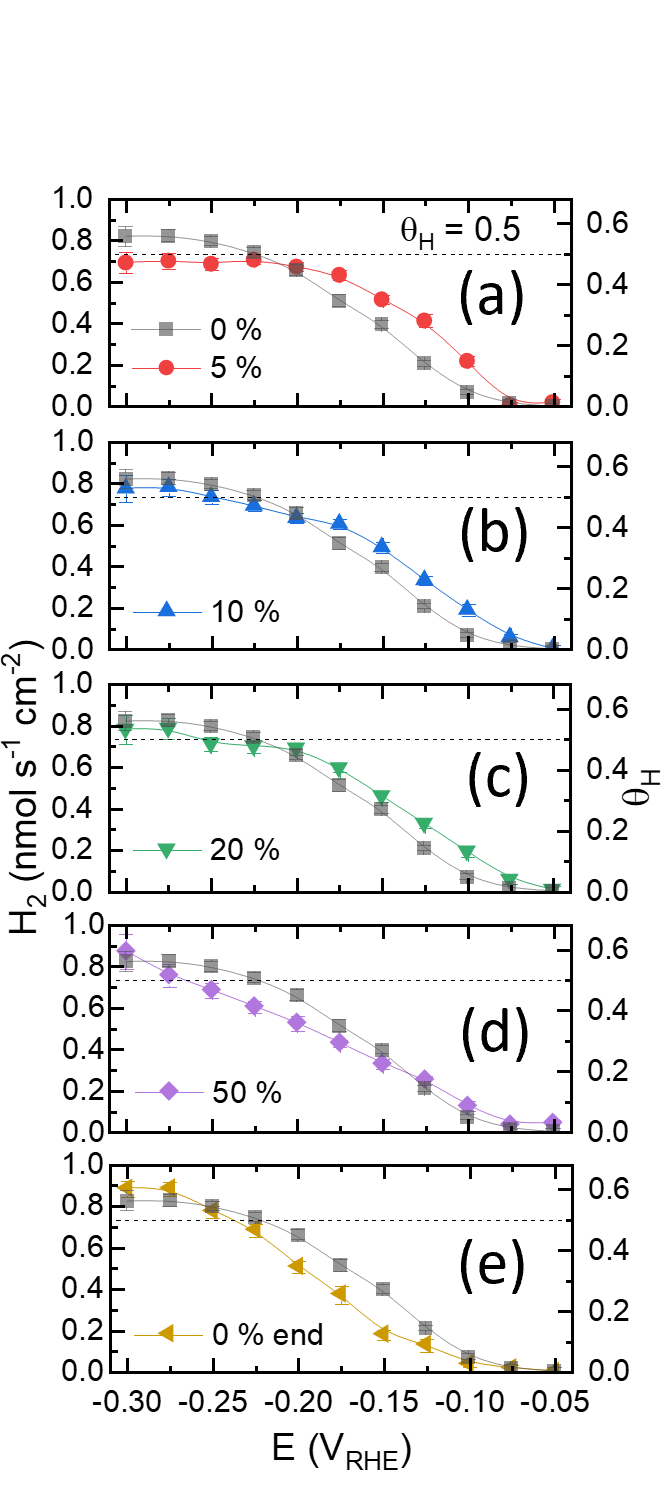


**Figure S26.** (a-e) Hydride coverage measured via recombination by mass spectrometry during potential pulse measurements on Cu(111) in 0.1 mol L^-1^ HClO_4_ with sequentially increasing O_2_ % in He, as indicated by the legends (complementary to **Figure 8**).


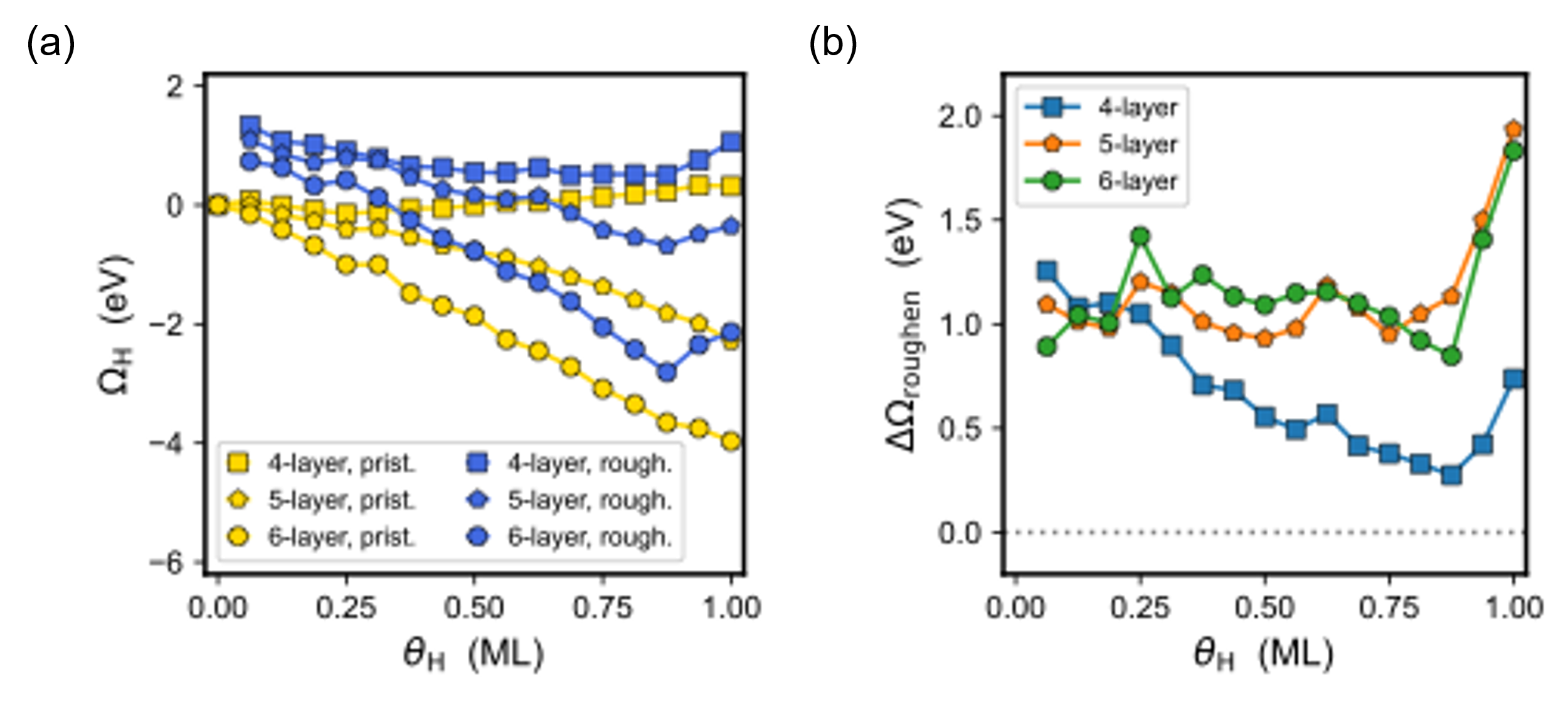


**Figure S27.** Test of the total number of atomic layers in the simulation slab. (a) Grand canonical free energies of pristine and roughened surface phases under varying H coverage at -0.25 V_RHE_ (as in main-text Figure 2), and (b) Grand canonical free energetics of surface roughening under varying H coverage, with 4, 5, and 6 atomic layers in the simulation slab. The metric measures the thermodynamic favorability for the hydride surfaces to self-roughen. The 4-layer model under-estimates the stability of higher coverage states, which would lead to a later hydride-forming onset, but the pristine-vs-roughened stability trend are qualitatively unchanged over the whole coverage regime. Therefore, the conclusion that the Cu surface roughening is thermodynamically unfavorable under pure H coverage (without O species) remains unchanged.


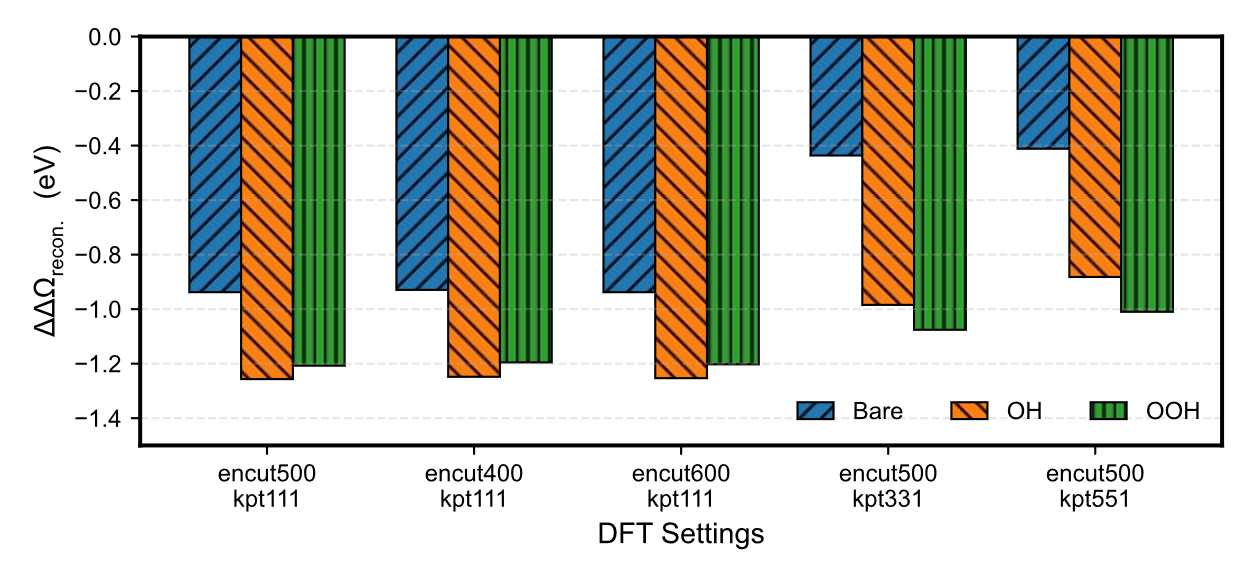


**Figure S28.** Test of different DFT settings on the relative grand canonical free energetics of surface roughening in absence or presence of OH and OOH adsorbates. The $\Delta\Delta\Omega_{recon.}$is the difference between the roughening energetics of metallic and hydride states as in main-text Figure 7a. The metric measures the facilitation of surface reconstruction by surface hydride and ORR intermediates. The X-axis labels represent the kinetic energy cutoff in units of eV (e.g., encut500 stands for 500 eV) and the k-points mesh (e.g., 331 stands for a 3X3X1)


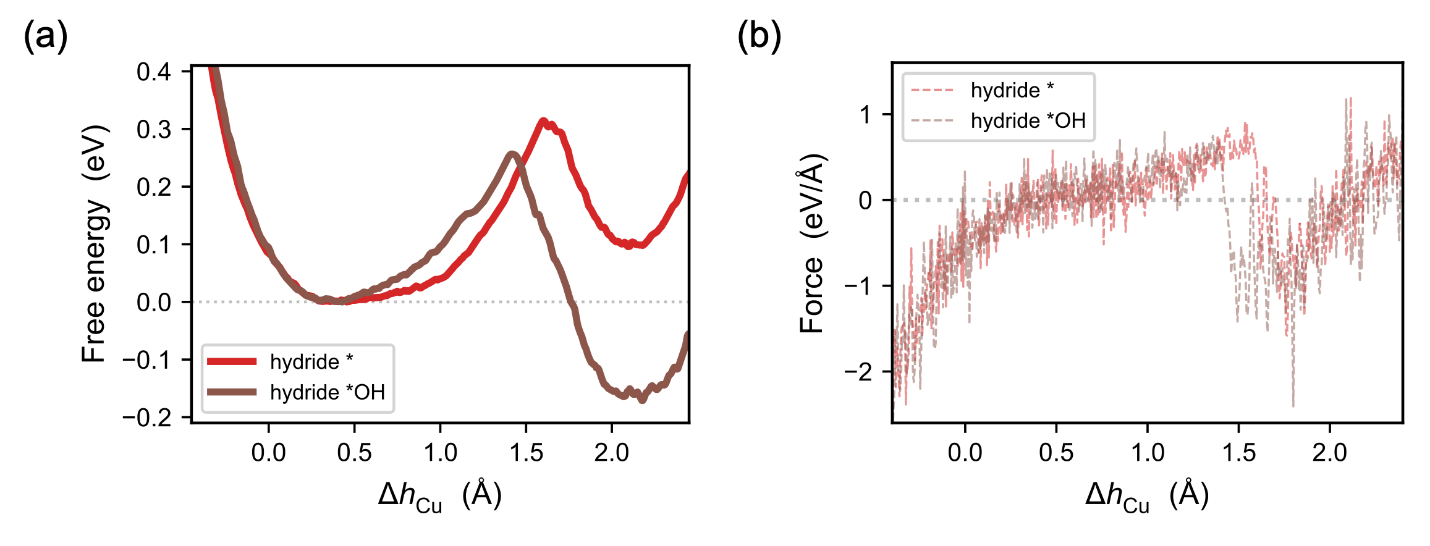


**Figure S29.** Free energy calculation of surface roughening at a tighter electronic convergence criterion of 10^-6^ eV. (a) The free energy profiles of surface Cu elevation on hydride surface with or without OH adsorbate. (b) The evolution of forces along the reaction coordinate during slow-growth MD simulations.

**Note**

*Certain commercial equipment, instruments, or materials are identified in this paper to specify the experimental procedure adequately. Such identification is not intended to imply recommendation or endorsement by the National Institute of Standards and Technology, nor is it intended to imply that the materials or equipment identified are necessarily the best available for the purpose.

**References**

(1) Tackett, B. M.; Raciti, D.; Hight Walker, A. R.; Moffat, T. P. Surface Hydride Formation on Cu(111) and Its Decomposition to Form H_2_ in Acid Electrolytes. *J. Phys. Chem. Lett.* **2021**, 10936–10941. https://doi.org/10.1021/acs.jpclett.1c03131.

(2) Raciti, D.; Moffat, T. P. Quantification of Hydride Coverage on Cu(111) by Electrochemical Mass Spectrometry. *J. Phys. Chem. C* **2022**, *126* (44), 18734–18743. https://doi.org/10.1021/acs.jpcc.2c06207.

(3) Krempl, K.; Hochfilzer, D.; Scott, S. B.; Kibsgaard, J.; Vesborg, P. C. K.; Hansen, O.; Chorkendorff, I. Dynamic Interfacial Reaction Rates from Electrochemistry–Mass Spectrometry. *Anal. Chem.* **2021**, *93* (18), 7022–7028. https://doi.org/10.1021/acs.analchem.1c00110.

(4) Trimarco, D. B.; Scott, S. B.; Thilsted, A. H.; Pan, J. Y.; Pedersen, T.; Hansen, O.; Chorkendorff, I.; Vesborg, P. C. K. Enabling Real-Time Detection of Electrochemical Desorption Phenomena with Sub-Monolayer Sensitivity. *Electrochimica Acta* **2018**, *268*, 520–530. https://doi.org/10.1016/j.electacta.2018.02.060.
